# Hyperleptinemia in obese state renders luminal breast cancers refractory to tamoxifen by coordinating a crosstalk between Med1, miR205 and ErbB

**DOI:** 10.1038/s41523-021-00314-9

**Published:** 2021-08-13

**Authors:** Arumugam Nagalingam, Sumit Siddharth, Sheetal Parida, Nethaji Muniraj, Dimiter Avtanski, Panjamurthy Kuppusamy, Justin Elsey, Jack L. Arbiser, Balázs Győrffy, Dipali Sharma

**Affiliations:** 1 Department of Oncology, Johns Hopkins University School of Medicine and the Sidney Kimmel Comprehensive Cancer Center at Johns Hopkins, Baltimore, MD USA; 2 Department of Medicine, University of Maryland, Baltimore, MD United States; 3 Department of Dermatology, Emory School of Medicine, Atlanta Veterans Administration Medical Center, Atlanta, GA USA; 4 MTA TTK Momentum Cancer Biomarker Research Group, Budapest, Hungary; 5 Semmelweis University, Department of Bioinformatics and 2nd Department of Pediatrics, Budapest, Hungary; 6 Present Address: Division of Endocrinology, Department of Medicine, Lenox Hill Hospital, New York, NY USA

**Keywords:** Cancer therapeutic resistance, Breast cancer

## Abstract

Obese women with hormone receptor-positive breast cancer exhibit poor response to therapy and inferior outcomes. However, the underlying molecular mechanisms by which obesity/hyperleptinemia may reduce the efficacy of hormonal therapy remain elusive. Obese mice with hyperleptinemia exhibit increased tumor progression and respond poorly to tamoxifen compared to non-obese mice. Exogenous leptin abrogates tamoxifen-mediated growth inhibition and potentiates breast tumor growth even in the presence of tamoxifen. Mechanistically, leptin induces nuclear translocation of phosphorylated-ER and increases the expression of ER-responsive genes, while reducing tamoxifen-mediated gene repression by abrogating tamoxifen-induced recruitment of corepressors NCoR, SMRT, and Mi2 and potentiating coactivator binding. Furthermore, in silico analysis revealed that coactivator Med1 potentially associates with 48 (out of 74) obesity-signature genes. Interestingly, leptin upregulates Med1 expression by decreasing miR-205, and increases its functional activation via phosphorylation, which is mediated by activation of Her2 and EGFR. It is important to note that Med1 silencing abrogates the negative effects of leptin on tamoxifen efficacy. In addition, honokiol or adiponectin treatment effectively inhibits leptin-induced Med1 expression and improves tamoxifen efficacy in hyperleptinemic state. These studies uncover the mechanistic insights how obese/hyperleptinemic state may contribute to poor response to tamoxifen implicating leptin-miR205-Med1 and leptin-Her2-EGFR-Med1 axes, and present bioactive compound honokiol and adipocytokine adiponectin as agents that can block leptin’s negative effect on tamoxifen.

## Introduction

Obesity is associated with increased risk of multiple types of cancers and notably, accounts for ~55% of all cancers diagnosed in women, including breast cancer^1^. Multiple epidemiological studies have shown that high body-mass-index (BMI) positively associates with larger tumors, more involved axillary nodes, increased metastatic progression, higher risk of recurrence and worse overall survival though the magnitude of impact may vary based on menopausal status, age, ethnicity as well as breast cancer subtype^2^. Importantly, women with hormone receptor-positive/ Her2-negative breast cancer show a continuous relation between worst outcomes and increasing BMI even after hormonal therapy^3^. It is imperative to understand the underlying molecular networks that may explain why women in the highest quintile of BMI exhibit double the breast cancer-related mortality rate in comparison to women in the lowest quintile. Obese state may be a surrogate for other oncogenic factors that not only contribute to breast cancer growth and progression but also interfere with responsiveness to endocrine therapy.

Accumulation of excess fat mass owing to the hypertrophy and hyperplasia of adipocytes, which are not only the energy storing cells but also active endocrine organs secreting adipocytokines, results in dysregulation of adipocytokines in obesity^4^. Hyperleptinemia (high levels of adipocytokine leptin) is an important manifestation of obese state and has been shown to mediate myriad biological impacts of obesity including carcinogenesis^5^. Higher serum leptin levels correlate with an increased risk of breast cancer as well as poor clinocopathological tumor characteristics in postmenopausal breast cancer patients^6,7^. Immunohistochemical analysis of breast tumors, peritumoral and adjacent normal breast tissues show elevated expression of leptin receptor and leptin in 83 and 92% of breast tumors, respectively, while adjacent normal breast tissue exhibit weak positivity^8,9^. Also, higher leptin and leptin receptor expression is observed in primary and metastatic invasive ductal breast carcinoma in comparison to normal breast tissue^10^. Previous studies from our group and others have shown the oncogenic effects of leptin on breast cancer^11–15^. High-fat-diet induced obesity in MMTV-TGF-α mice results in elevated leptin levels as well as shorter latency and higher mammary tumor load^16^. Exogenous leptin administration increases tumor growth in athymic nude mice^13^. Upon leptin exposure, breast cancer cells exhibit increased proliferation, reduced apoptosis, and enhanced angiogenesis to support greater tumor growth^17,18^. Leptin also supports metastatic progression and tumor recurrence by increasing invasion and migration potential as well as stem-like characteristics in breast cancer cells^19^. However, despite these advances, it is unclear how obesity/hyperleptinemia may reduce the efficacy of hormonal therapy.

In this study, we provide evidence that obese hyperleptinemic mice not only exhibit higher breast tumor burden in comparison to lean mice but are also refractory to tamoxifen treatment forming the rationale of our work that the elevated levels of leptin in obese state may modulate the underlying signaling mechanisms to block tamoxifen. Significantly, our studies show that leptin induces ER phosphorylation and transcriptional activation in a ligand-independent manner to abrogate tamoxifen-mediated gene repression. Leptin potentiates the release of corepressors and recruitment of coactivators to ER-responsive gene promoters in tamoxifen-treated breast cancer cells. We present Med1, an important subunit of the human mediator complex, as the key node of leptin action that associates with obesity-signature genes. Subsequent mechanistic studies show that leptin orchestrates Med1 activation via decreasing miR-205 and increasing upstream kinases Her2 and EGFR phosphorylation. Moreover, we show that Med1 silencing inhibits leptin’s negative effects on tamoxifen. We also present bioactive compound honokiol and adipocytokine adiponectin as potential agents to improve tamoxifen efficacy in hyperleptinemia.

## Results

### Hyperleptinemia diminishes the anti-cancer effect of tamoxifen

**Fig. 1:**
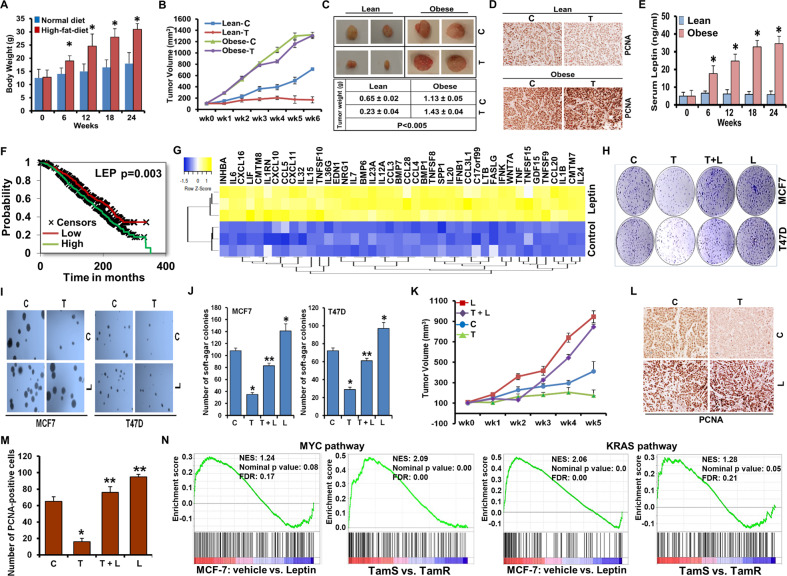
Obese hyperleptinemic state associates with decreased effectiveness of tamoxifen. **A** Mice were fed high-fat-diet (HFD) or control normal-diet (ND) to achieve obese/lean phenotype. Body weights of mice were measured after 6, 12, 18, and 24 weeks of diet intervention (*n* = 24). *p < 0.05, compared with ND mice. **B** MCF7 cells-derived tumors were developed in obese and lean mice and treated with 4-hydroxytamoxifen (T) or vehicle (C). *p < 0.05. **C** Representative tumor pictures and average tumor weights from obese and lean mice treated with tamoxifen (T) or vehicle (C) are shown. **D** Expression of PCNA was examined using immunohistochemistry. Representative images are included. Scale bar: 50 µm. **E** Serum samples from HFD/obese or ND/lean mice were subjected to ELISA to quantify leptin levels at indicated time intervals. *p < 0.05, compared with untreated controls. **F** Kaplan–Meier analyses for overall survival of the cohort of patients with ER-positivity, receiving tamoxifen treatment with chemotherapy in relation to mean leptin (LEP) expression (*p* = 0.003). **G** Upregulated expression of a set of cytokine pathway genes was observed in MCF7 cells treated with leptin. **H** MCF7 and T47D cells were treated with 100 ng/ml leptin (L) and/or 1 μM 4-hydroxytamoxifen (T) as indicated and anchorage-dependent growth was examined by clonogenicity assay. Vehicle-treated cells are denoted with (**C**). **I**, **J** Soft-agar colony-formation of MCF7 and T47D cells treated with leptin (L) and/or 4-hydroxytamoxifen (T) as in H for 3 weeks. Histogram represents average number of colonies counted (in six micro-fields). **p < 0.005, *p < 0.05. **K** MCF7 cells-derived tumors were developed in athymic nude mice and treated with vehicle (C), Leptin (L), 4-hydroxytamoxifen (T) or 4-hydroxytamoxifen+Leptin (T+L). Tumor growth was monitored by measuring the tumor volume for 5 weeks. (*n* = 8–10); (**p < 0.005). **L**, **M** Tumors from vehicle (C), Leptin (L), 4-hydroxytamoxifen (T) or T+L-treated mice were subjected to immunohistochemical (IHC) analysis using PCNA antibodies. Scale bar: 50 µm. Histogram shows number of PCNA positive cells. **p < 0.005, *p < 0.05. **N** GSEA showing a positive enrichment of MYC and KRAS pathways in leptin-treated and tamoxifen-resistant MCF7 cells. NES, normalized enrichment score. Data are means ± SD from three experiments.

Inferior disease-free and overall survival observed in obese women with breast cancer is often attributed to poor response to standard therapeutic regimens including endocrine therapy but mechanistic links are unknown. Hence, we queried whether diet-induced obesity (DIO) is capable of impacting the efficacy of tamoxifen in breast cancer. We employed the orthotopic CDX (cell line-derived xenograft) model in obese and non-obese NOD/SCID mice. An obese phenotype was generated using high-fat diet (HFD; 45% kcal fat diet). Obese-mice weighed significantly more than lean-mice (Normal-diet; 10% low-fat control diet) (Fig. 1A). Obese-mice exhibited increased tumor progression and significantly larger tumors compared to lean-mice. Interestingly, obese-mice showed an increase in the tumor progression and tumor load upon tamoxifen treatment and were comparable to vehicle-treated obese-mice whereas lean-mice showed effective tumor regression in response to tamoxifen (Fig. 1B, C). Analysis of tumor sections showed higher expression of PCNA in obese-vehicle as well as obese-tamoxifen group in comparison to tamoxifen-treated lean-mice (Fig. 1D). Hallmarks of obesity include adipocytokine dysregulation where hyperleptinemia (high leptin levels) is closely tied with higher BMI. Indeed, obese-mice exhibited significantly higher circulating leptin levels in comparison to lean-mice (Fig. 1E). Then we analyzed whether the leptin gene expression itself correlated with overall survival in a cohort of ER+ breast cancer patients. Kaplan–Meier analysis indicated that elevated expression of leptin associated with poor overall survival in breast cancer patients (*p* = 0.003) with estrogen receptor (ER) positive breast cancer (Fig. 1F). In silico analyses of differentially expressed genes (DEGs) in MCF7 cells treated with vehicle vs. leptin^20^ showed increased levels of several cytokines indicating the importance of leptin as an upstream adipokine (Fig. 1G). We also observed differential regulation of several pathways related to receptor activity (Supplementary Figs 1, 2), estrogen response genes (Supplementary Fig. 3) and EGFR signaling pathway (Supplementary Fig. 4). Collectively, several important genes/pathways are differentially modulated upon leptin treatment. Next, we examined the effect of hyperleptinemia on tamoxifen-mediated growth inhibition of breast cancer cells. Tamoxifen treatment inhibited clonogenic potential (~70% inhibition) and anchorage-independent growth of breast cancer cells as expected but co-exposure of cells with leptin abrogated tamoxifen’s growth-inhibitory effects (Fig. 1H–J). Encouraged by our in vitro findings we investigated whether leptin exposure impacts tamoxifen-mediated tumor inhibition. More rapid tumor progression was observed in leptin-treated group compared to control-group while tamoxifen treatment regressed tumors effectively. Interestingly, mice treated with tamoxifen and leptin exhibited higher tumor growth similar to leptin alone group showing that leptin exposure renders breast tumors unresponsive to tamoxifen (Fig. 1K). Immunohistochemical analysis showed higher PCNA nuclear staining in leptin-treated tumors. Tumors from mice treated with leptin and tamoxifen also exhibited higher PCNA staining in comparison to tamoxifen alone group (Fig. 1L–M) indicating higher proliferation. Interestingly, gene set enrichment analysis (GSEA) of the DEGs in MCF7 cells treated with vehicle vs. leptin^20^ or tamoxifen-sensitive vs. tamoxifen-resistant MCF7 cells^21^ showed a positive enrichment of similar tumor growth-associated pathways (MYC and KRAS) in leptin-treated MCF7 cells as well as tamoxifen-resistant MCF7 cells (Fig. 1N). Overall, these results show that hyperleptinemia associated with obesity significantly reduces the efficacy of tamoxifen in ER-positive breast cancer cells.

### Leptin exerts ligand-independent activation of estrogen receptor and abrogates tamoxifen-mediated recruitment of corepressors

**Fig. 2:**
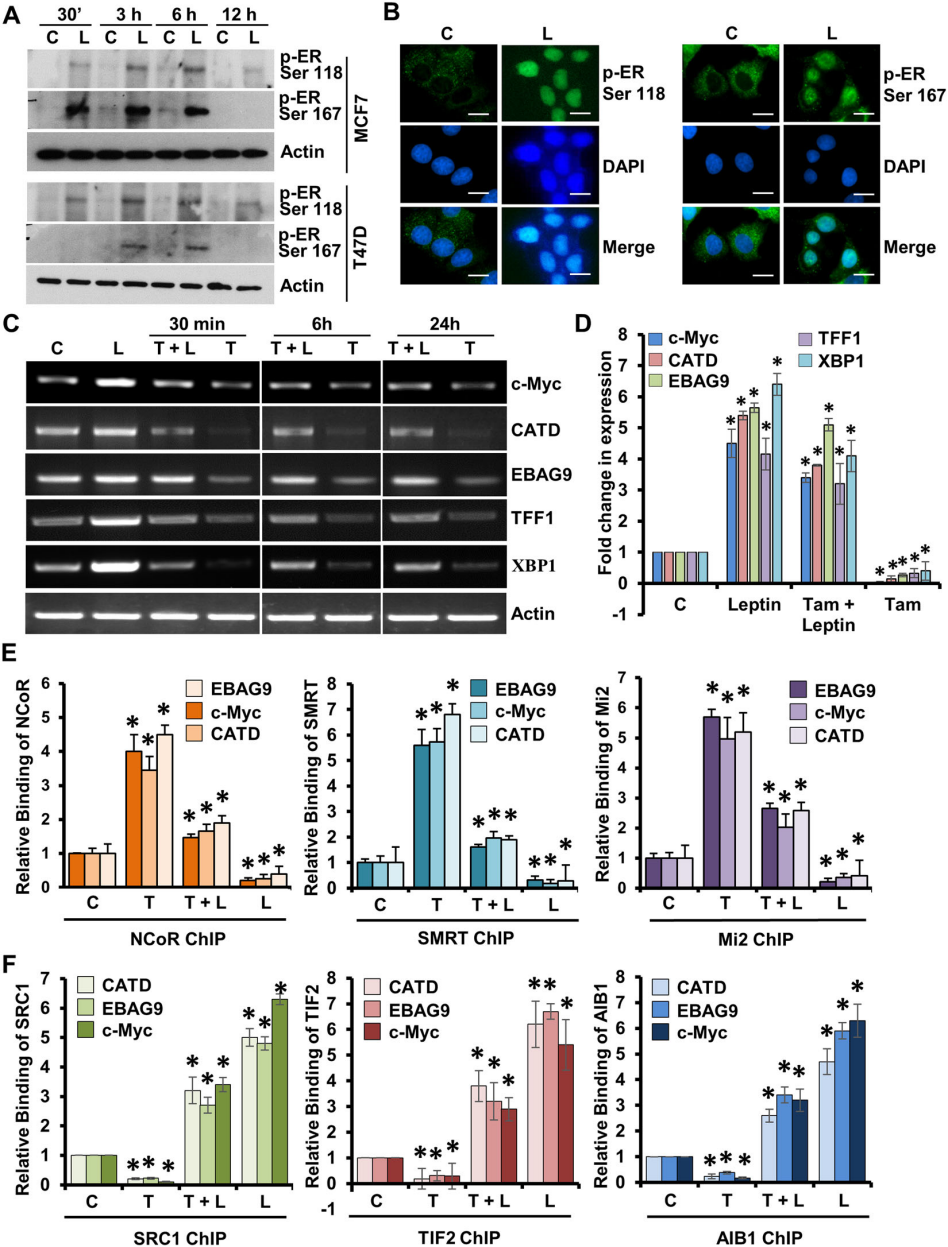
Leptin activates estrogen receptor and abrogates tamoxifen-mediated recruitment of corepressors. **A** MCF7 and T47D cells were grown in dextran charcoal stripped media and phenol red free media prior to stimulation with leptin. Cells were treated with 100 ng/ml leptin for indicated time-intervals and subjected to immunoblot analysis for phosphorylated estrogen receptor proteins as denoted. Actin was used as control. **B** MCF7 cells were treated with 100 ng/ml leptin followed by immunocytochemical analysis and confocal imaging. Scale bar: 10 µm. **C**, **D** Semi-quantitative RT-PCR and real-time quantitative PCR analyses of mRNA expression levels of c-Myc, Cathepsin D (CATD), EBAG9, TFF1, and XBP1 in MCF7 cells treated with 100 ng/ml leptin (L) and/or 1 μM 4-hydroxytamoxifen (T) as indicated. **E** Chromatin immunoprecipitation assays were performed using antibodies specific for NCoR, SMRT and Mi2 in MCF7 cells treated with 100 ng/ml leptin (L) and/or 1 μM 4-hydroxytamoxifen (T). The purified DNA was analyzed by PCR using specific primers spanning the EREs of EBAG9, cathepsin D (CATD) and c-myc gene promoters. *p < 0.05. **F** Chromatin immunoprecipitation assays were performed using antibodies specific for SRC1, TIF2, and AIB1 in MCF7 cells treated with 100 ng/ml leptin (L) and/or 1 μM 4-hydroxytamoxifen (T). The purified DNA was analyzed by PCR using specific primers spanning the EREs of EBAG9, cathepsin D (CATD) and c-myc gene promoters. *p < 0.05. Data are means ± SD from three experiments.

Ligand binding or activation of upstream kinase pathways enhances the phosphorylation of estrogen receptor α (ER) at multiple sites leading to its functional activation^22^. Upon activation, ER undergoes nuclear translocation, and is recruited to the ER-responsive elements (EREs) on the promoters of ER-responsive genes resulting in increased gene expression^23^. We questioned whether leptin exposure affects phosphorylation and nuclear translocation of ER in breast cancer cells. MCF7 and T47D cells, treated with leptin, showed increased phosphorylation of ER at Ser118 and Ser167 (Fig. 2A). Immunofluorescence analysis with antibodies specific for phosphorylated ER-Ser118 or ER-Ser167 revealed increased nuclear translocation of phosphorylated estrogen receptor upon leptin treatment (Fig. 2B). As expected, tamoxifen treatment decreased the expression of c-Myc, Cathepsin D, EBAG9, TFF1 and XBP1 genes. Leptin exposure, on the other hand, not only increased the expression of ER-responsive genes, but also mitigated tamoxifen-induced gene repression (Fig. 2C, D). Tamoxifen-bound ER undergoes a conformational alteration and preferentially recruits corepressor complexes to the EREs, and inhibits the expression of ER-responsive genes^23^. Leptin exposure resulted in minimal corepressor binding on the EREs while tamoxifen treatment stimulated increased recruitment of NCoR, SMRT and Mi2 in a chromatin immunoprecipitation assay. Interestingly, leptin exposure significantly reduced the recruitment of NCoR, SMRT and Mi2 on the EREs of EBAG9, c-Myc and Cathepsin D genes in the presence of tamoxifen (Fig. 2E). In contrast, leptin treatment stimulated the recruitment of coactivators SRC1, TIF2 and AIB1 on the EREs of EBAG9, c-Myc, and Cathepsin D genes whereas tamoxifen treatment inhibited the coactivator binding. Leptin treatment also resulted in coactivator recruitment in the presence of tamoxifen (Fig. 2F). These results present that leptin exerts ligand-independent activation of estrogen receptor and rescues expression of ER-responsive genes from tamoxifen-mediated inhibition via reducing the recruitment of corepressors and increasing the coactivator binding.

### Leptin induces upregulation of coactivator Med1 via inhibition of miR205

**Fig. 3:**
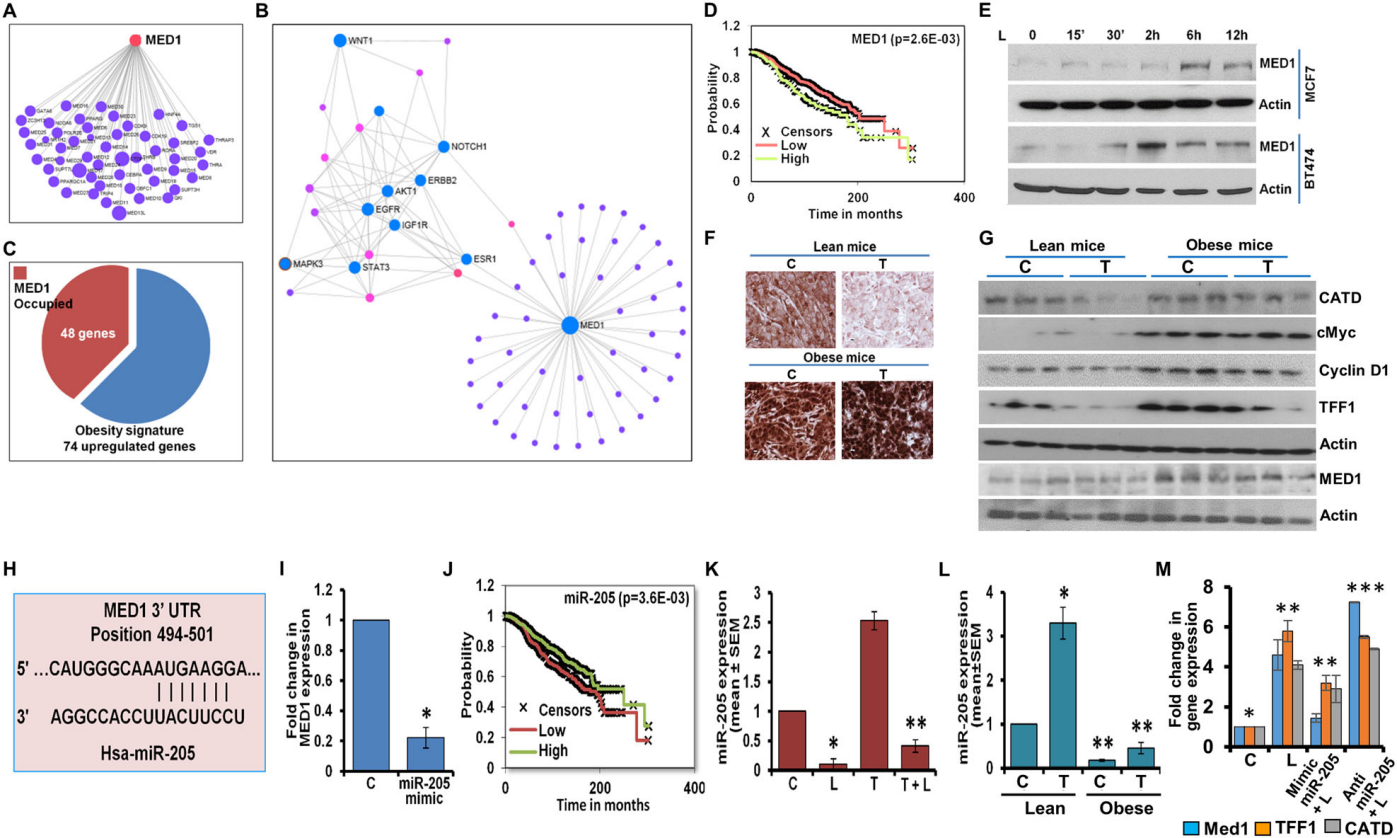
Med1, an important node associated with leptin signaling network and obesity-associated signature in breast cancer, is upregulated via miR-205 in hyperleptinemic state. **A** Protein-protein interaction landscape of Med1 and mediator complex subunits and associated proteins is presented. **B** An integrated network of proteins involved in leptin signaling network and Med1 is presented. In total, ~70 proteins are represented, 9 of which serve as nodes in the Med1-leptin interaction network. Blue circles represent high confidence leptin-signaling proteins. Pink and purple circles represent additional proteins identified in Med1 protein-protein interaction network. To avoid the overcrowded network, only selected proteins are represented. **C** Obesity-related gene signature was queried in Med1 ChIP-seq data. 48 out of 75 genes upregulated in breast tumors in obese patients are potential targets of Med1. A summary of Med1 associated proteins that associate with leptin is presented in Supplemental Table S1. **D** Kaplan–Meier plots of breast cancer survival in relation to mean Med1 (PPARBP) expression (*p* = 2.6E–03). **E** MCF7 and BT474 cells were treated with 100 ng/ml leptin (L) for various time intervals as indicated and immunoblotted for Med1 expression. **F**, **G** MCF7 cells derived tumors from high-fat-diet (HFD) fed obese mice and normal-diet (ND) fed lean mice and treated with 4-hydroxytamoxifen (T) or vehicle (C) were subjected to immunohistochemical (IHC) using Med1 antibody (Scale bars: 50 µm) and immunoblot analysis using Cathepsin D (CATD), cMyc, Cyclin D1, TFF1, and Med1, antibodies. Actin was used as loading control in immunoblot analysis. **H** The miRNA target pairing sequences were analyzed by bioinformatics tools. **I** MCF7 cells were transfected with miR-205 mimic and Med1 expression was examined using real-time PCR. **J** Kaplan–Meier plots of breast cancer survival in relation to mean miR-205 expression (*p* = 3.6E–03). **K** Expression of miR-205 was examined in MCF7 cells treated with 100 ng/ml leptin (L) and/or 1 μM 4-hydroxytamoxifen (T) as indicated. (**L**) Expression of miR-205 was examined in MCF7 cells-derived tumors from high-fat-diet (HFD) fed obese mice and normal-diet (ND) fed lean mice and treated with 4-hydroxytamoxifen (T) or vehicle (C). **M** MCF7 cells were transfected with miR-205-mimic and anti-miR-205 followed by treatment with 100 ng/ml leptin (L). Med1, TFF1 and CATD expression was examined using quantitative real-time PCR. ***p < 0.0005, **p < 0.005, *p < 0.05. Data are means ± SD from three experiments.

Transcriptional activation by ER involves the recruitment of various coactivators that coordinate chromatin remodeling and facilitate the assembly of RNA polymerase II complex^23^. Mediator complex has emerged as the key coactivator complex that interacts with ER through Med1^24^. Med1 is a key coactivator responsible for adipogenesis^25^ and it has also been associated with ER-transactivation^24^. Protein-protein interaction analysis using Cytoscape showed that Med1 itself was a major hub that directly interacted with diverse subunits of the mediator complex and beyond (Fig. 3A). Interestingly, highly integrated protein-protein interaction landscape of Med1 involved key leptin signaling pathways including its canonical (Akt, Stat3, and ERK) as well as non-canonical (Wnt, Notch, IGF1R and ErbB kinases) nodes (Fig. 3B). Breast tumors in obese state harbor a distinct obesity-associated gene transcription signature (GSE24185)^26^ consisting of 725 downregulated probes and 74 probes that are upregulated with high BMI. Since Med1 is a transcriptional coactivator, we queried whether obesity-associated upregulated-genes in breast cancer are transcriptional targets for Med1. Using Med1 ChIP-seq data from ChIP-Atlas (https://chip-atlas.org/), we noted that among the 74 genes upregulated in obesity-breast cancer signature, 48 genes were putative targets for Med1 recruitment (Fig. 3C, Supplementary Table 1), indicating its importance in obesity-breast cancer connection. Indeed, a strong association between higher Med1 expression and poor overall survival was observed in breast cancer patients with estrogen receptor positive breast cancer (Fig. 3D). Next, we investigated whether obese state and hyperleptinemia influence Med1 expression in breast cancer. Breast cancer cells exposed to leptin showed a temporal increase in Med1 expression (Fig. 3E). IHC analysis showed elevated expression of Med1 in obese state which remained elevated upon tamoxifen treatment (Fig. 3F). Increased expression of Cathepsin D (CATD), cMyc, Cyclin D1, TFF1 and Med1 was noted in MCF7 cells-derived tumors formed in obese-mice in comparison to lean-mice. In addition, their expression remained elevated in breast tumors in obese-mice treated with tamoxifen, whereas tumors in lean-mice exhibited lower expression in vehicle as well as tamoxifen-treated groups (Fig. 3G).

Various microRNAs (miRs) act as tumor suppressors or activators, controlling multiple aspects of tumor progression including tumor cell proliferation, apoptosis, invasion, migration and stemness, via modulating the expression of key genes^27^. We queried the involvement of miR(s) in modulation of Med1 expression in obese and hyperleptinemic state. Using bioinformatics algorithms (TargetScan, RNA22 and miRBase), we identified conserved target sites for miR-205 binding site at nucleotides 494–501 of the Med1 3ʹUTR (Fig. 3H). Ectopic expression of miR-205 (in the form of a mimic molecule) reduced the expression of Med1 (Fig. 3I). Breast cancer patients with estrogen receptor positive breast cancer showed a strong association between higher miR-205 expression and better overall survival (Fig. 3J) in contrast to Med1 whose overexpression associated with poor survival (Fig. 3D). Breast cancer cells exposed to leptin showed decreased levels of miR-205 whereas tamoxifen treatment significantly increased the expression of miR-205. Leptin exposure abrogated tamoxifen-mediated induction of miR-205 (Fig. 3K). Next, we examined whether obese state imparts similar effect on miR-205 expression. Indeed, MCF7 cells-derived tumors formed in vehicle-treated obese-mice showed lower miR-205 expression in comparison to vehicle-treated lean-mice. Tumors from tamoxifen-treated lean-mice exhibited higher levels of miR-205 expression whereas tamoxifen-treated obese-mice showed significant reduction in miR-205 level (Fig. 3L). We further examined the role of miR-205 in leptin-mediated activation of Med1 as well as ER-responsive genes. Breast cancer cells overexpressing miR-205 reduced leptin-mediated Med1, TFF1, and CATD overexpression whereas expression of miR-205-inhibitor potentiated the effect of leptin on Med1 expression (Fig. 3M). Taken together, these results show that Med1 and miR205 exhibit contrasting association with ER-positive breast cancer prognosis and leptin increases the expression of Med1, a key coactivator protein, by inhibiting the expression of miR-205.

### Leptin orchestrates phosphorylation, nuclear accumulation and promoter-recruitment of Med1 via activation of EGFR and Her2 kinases

**Fig. 4:**
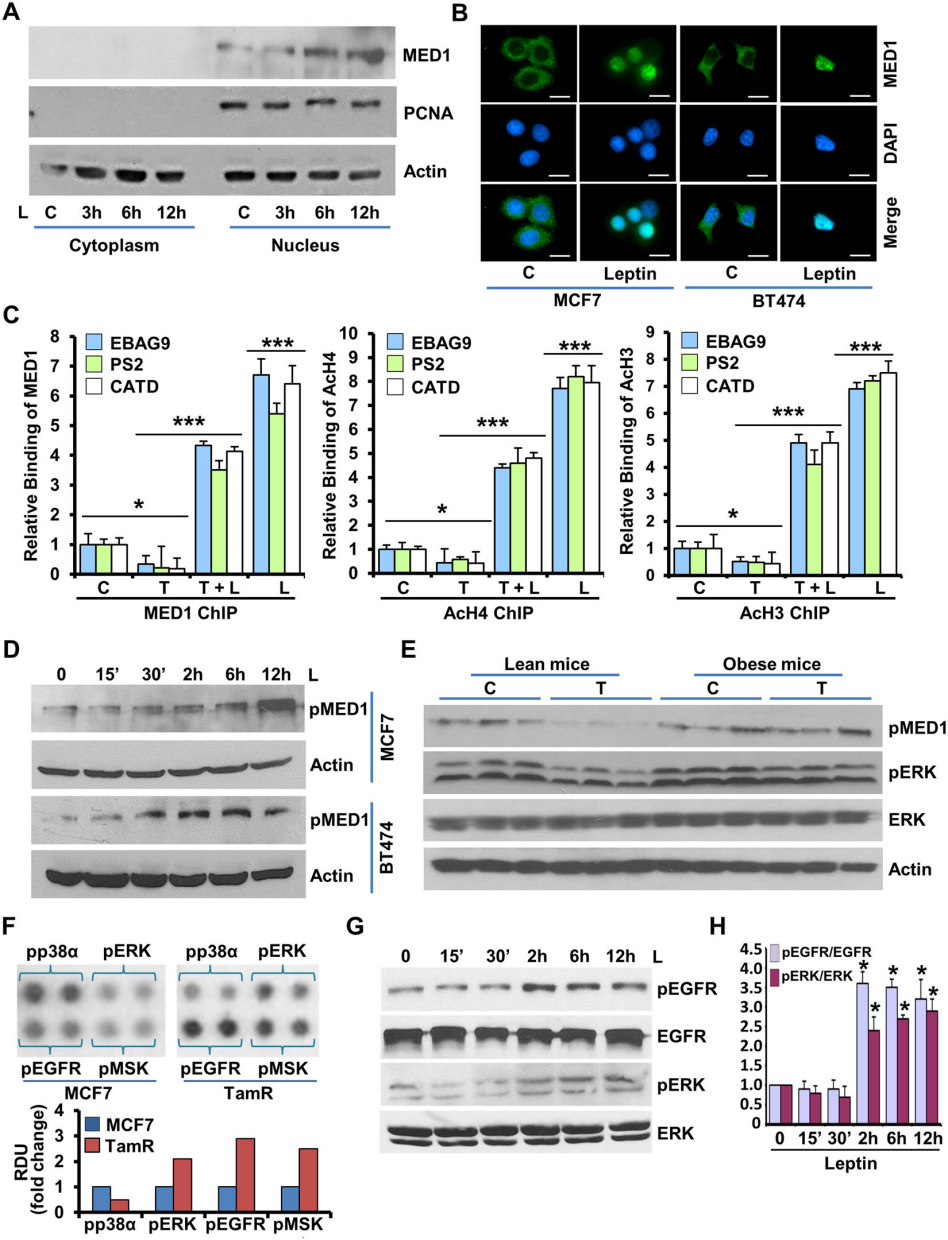
Leptin induces phosphorylation, nuclear accumulation and promoter-recruitment of Med1 in breast cancer. **A** Immunoblot analysis of Med1 in cytoplasmic and nuclear fractions of MCF7 cells treated with 100 ng/ml leptin (L). PCNA and actin were used as controls. **B** Immunofluorescence analysis of Med1 in MCF7 and BT474 cells treated with 100 ng/ml leptin (L). Scale bar: 10 µm. **C** MCF7 cells were treated with 100 ng/ml leptin (L) and/or 1 μM 4-hydroxytamoxifen (T) and chromatin immunoprecipitation assays were performed using antibodies specific for Med1, Ac-H4, and Ac-H3. The purified DNA was analyzed by PCR using specific primers spanning the EREs of EBAG9, PS2, and cathepsin D (CATD) gene promoters. **D** Total protein was isolated from MCF7 and BT474 cells followed by immunoblot analysis for phosphorylated-Med1 expression. Actin was used as control. **E** Immunoblot analysis of total protein isolated from MCF7 cells-derived tumors from high-fat-diet (HFD) fed obese mice and normal-diet (ND) fed lean mice and treated with 4-hydroxytamoxifen (T) or vehicle (C). Expression of pMed1, pERK and ERK was examined. Actin was used as control. **F** Protein lysates from MCF7 and TAM R cells subjected to human phospho-antibody array analyses. Bar diagram shows relative levels of protein phosphorylation as indicated (normalized intensity for each antibody) were calculated for each sample and compared between MCF7 and TAM R cells. **G** MCF7 cells were treated with 100 ng/ml leptin as indicated and total lysates were immunoblotted for pEGFR, EGFR, pERK and tERK expression levels. **H** Bar diagram shows the ratio of pEGFR/EGFR and pERK /tERK in breast cancer cells treated with leptin as indicated. *p < 0.05. Data are means ± SD from three experiments.

Med1 is a key coactivator protein for ER-mediated transcription;^24^ next, we queried whether leptin exposure affects the nuclear localization and coactivation function of Med1. Leptin exposure resulted in higher level of Med1, which was accumulated in nucleus (Fig. 4A, B). Earlier studies have shown that Med1 is essential for the expression of ER-dependent genes and estrogen mediated breast cancer growth^24,28^. While estrogen-activated ER recruits Med1 to facilitate histone acetylation on the promoter region of ER-responsive genes, tamoxifen-bound ER presents a steric hindrance to block coactivator recruitment^23,24^. As expected, breast cancer cells treated with tamoxifen showed minimal recruitment of Med1 on the EREs of ER-responsive genes as well as reduced levels of acetylated H3 and H4 binding. Leptin, on the other hand, stimulated significant increase in Med1 recruitment as well as H3/H4 binding on EBAG9, PS2, and CATD genes and also abrogated the inhibitory effects of tamoxifen on coactivator recruitment (Fig. 4C). Phosphorylation of Med1 not only potentiates its interaction with mediator complex, but also required for its transactivation function^29^. Interestingly, increased level of phosphorylated Med1 was observed in leptin-treated MCF7 and BT474 cells (Fig. 4D). Furthermore, immunoblot analysis of MCF7 cells-derived tumors formed in obese mice treated with vehicle or tamoxifen exhibited higher expression of phosphorylated-Med1 in comparison to tamoxifen-treated lean mice (Fig. 4E). Since our results indicated that leptin exposure promotes a tamoxifen-refractory breast cancer cell phenotype, we investigated the kinases involved in tamoxifen-resistant breast cancer model to further understand the mechanistic underpinnings. Utilizing a phosphoprotein array comprising of 46 specific Ser/Thr/Tyr phosphorylation sites of 38 selected proteins, we found elevated levels of phosphorylated ERK, EGFR and MSK in tamoxifen resistant breast cancer cells (Fig. 4F). Showing parallels, an increased level of phosphorylated ERK was observed in tumors from obese mice treated with vehicle or tamoxifen (Fig. 4E). Also, elevated levels of phosphorylated EGFR and ERK were observed in leptin treated breast cancer cells compared to untreated cells (Fig. 4G, H).

**Fig. 5:**
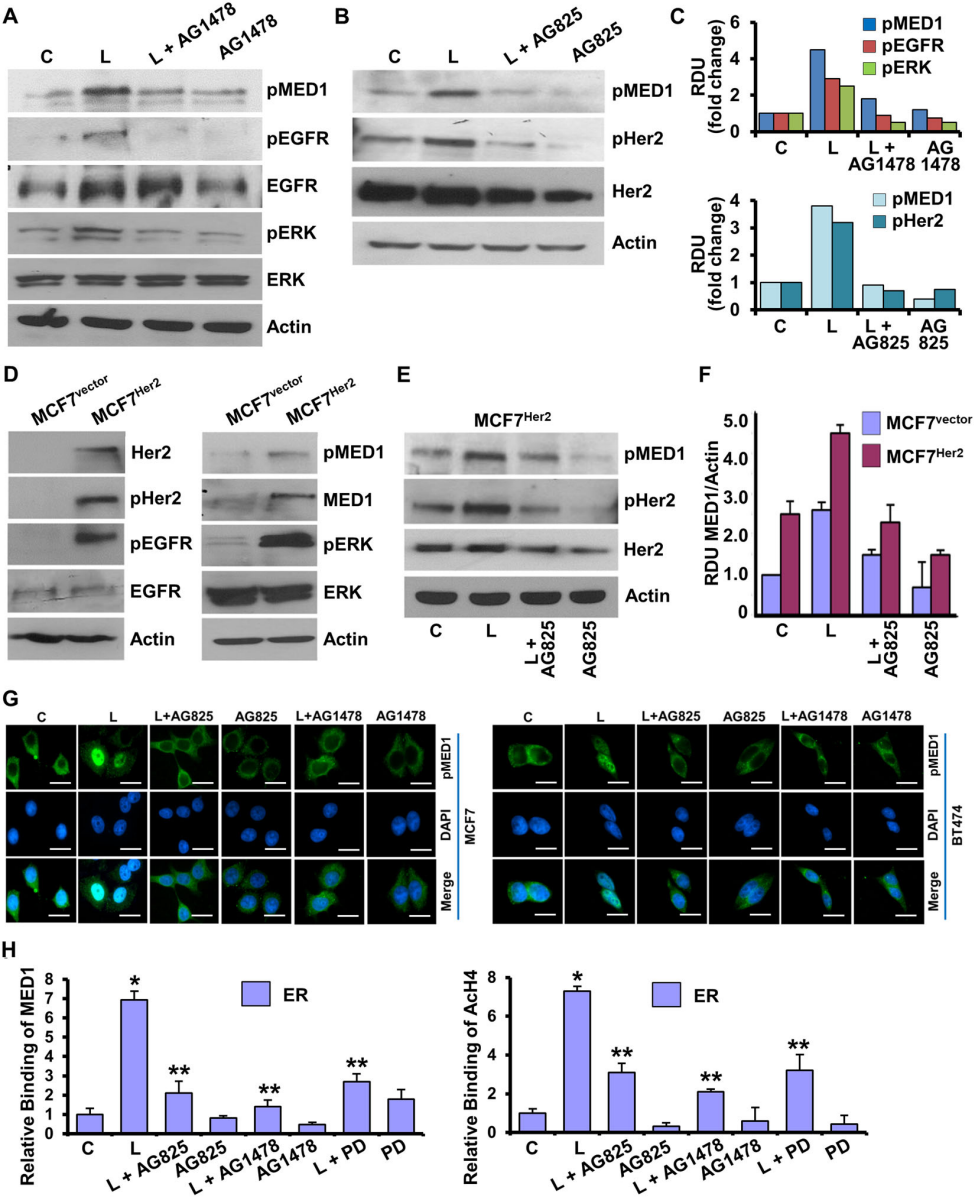
ERbB kinase inhibition abrogates activation of MED1 and recruitment of MED1 to ER promoter in response to leptin. **A** BT474 cells were treated with Leptin and AG1478 alone or in combination for 24 h. Cell lysates were immunoblotted with indicated antibodies. **B** BT474 cells were treated with Leptin and AG825 alone or in combination for 24 h. Cell lysates were immunoblotted for pMed1, pHER2, and tHER2. Actin was used as loading control. **C** Bar graph shows fold change in protein expression in (**A**) and (**B**). **D** Immunoblot analysis of MCF7-vector and MCF7-Her2 O/E cells for indicated proteins. **E**, **F** MCF7-HER2 O/E cells were treated with Leptin, AG825 alone or in combination for 24 h. Cell lysates were immunoblotted for pMED1, pHER2, and tHER2. Actin was used as loading control. Bar graph shows the ratio of MED1 and actin. **G** MCF7 and BT474 cells were treated as in (**A**) and (**B**), and subjected to immunofluorescence analysis of pMED1. Scale bar, 10 µm. **H** MCF7 cells were treated as indicated for 24 h and subjected to ChIP assay using Med1 antibody. The purified DNA was analyzed by using specific primer for ER promoter. **p < 0.005, *p < 0.05. Data are means ± SD from three experiments.

Further exploration of the direct involvement of Her2 and EGFR in leptin-mediated Med1 phosphorylation showed increased activation of EGFR, ERK, and Her2 in leptin treated breast cancer cells (Fig. 5A–C). Combined treatment with leptin and EGFR inhibitor, AG1478, inhibited leptin-induced Med1 phosphorylation in comparison to breast cancer cells treated with leptin alone (Fig. 5A, C). In addition, inhibition of Her2 activation with AG825, a selective ATP-competitive inhibitor of the tyrosine kinase activity of HER2/Neu, also abrogated leptin-induced Med1 phosphorylation (Fig. 5B, C). MCF7 cells stably overexpressing Her2 (MCF7^Her2^) not only exhibited increased activation of Her2, EGFR and ERK, but also showed increased phosphorylation of Med1 compared to MCF7-vector control cells, indicating the direct involvement of Her2 in Med1 phosphorylation (Fig. 5D). Leptin treatment further increased Med1 phosphorylation in MCF7^Her2^ cells, which was inhibited upon treatment with AG825 in combination with leptin (Fig. 5E, F). Next, we evaluated whether inhibiting upstream kinases impacts leptin-induced nuclear localization of Med1. As expected, immunofluorescence analysis of MCF7 and BT474 cells treated with leptin showed increased nuclear accumulation of Med1 compared to untreated cells. Inhibition of EGFR and Her2 with AG1478 and AG825 treatment alleviated the nuclear localization of Med1 in leptin-exposed cells (Fig. 5G). As Med1 phosphorylation is known to impact its transactivation function, we explored the involvement of EGFR, Her2 and ERK kinases in the recruitment of Med1 to ER promoter. ER itself is an estrogen-responsive gene and its promoter harbors a functional ERE^30^. Chromatin immunoprecipitation analysis showed that leptin induced the recruitment of Med1 on ERE region of ER promoter while co-treatment of breast cancer cells with AG825, AG1478, or PD98059 along with leptin resulted in significant reduction in Med1 binding (Fig. 5H). These results explicitly show that leptin induces phosphorylation, nuclear accumulation, promoter recruitment of Med1 in breast cancer cells and inhibition of ERbB kinases abrogates leptin-stimulated Med1 phosphorylation as well as its transactivation function.

### Med1 inhibition using adipocytokine adiponectin and bioactive Honokiol effectively improves the efficacy of tamoxifen in hyperleptinemic state

**Fig. 6:**
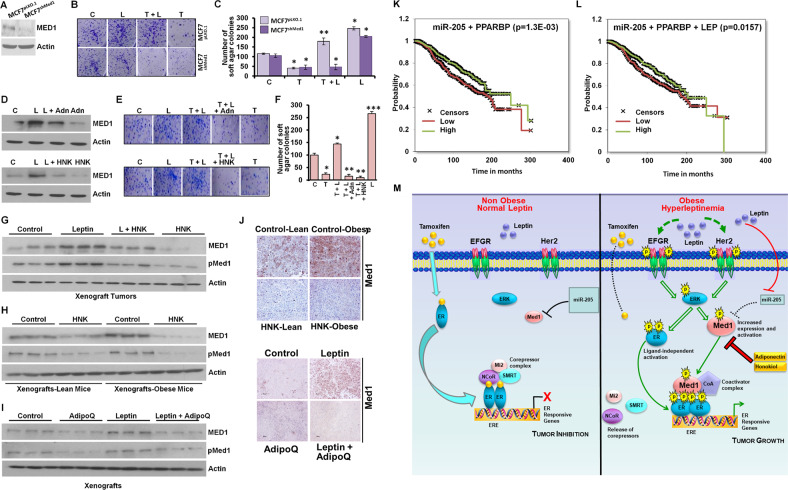
Bioactive strategies effectively improve the efficacy of tamoxifen in hyperleptinemic state. **A** Immunoblot analysis of Med1 expression level in stable pools of Med1-depleted (Med1^shRNA^) and vector control (pLKO.1) MCF7 cells. **B** Clonogenicity assay performed with MCF7-pLKO.1, MCF7 -pMed1^shRNA^ cells in the presence of leptin and 4-hydroxytamoxifen alone or with combination. **C** Soft agar assay was performed using MCF7-pLKO.1, MCF7 -pMed1^shRNA^ cells in the presence of leptin and 4-hydroxytamoxifen alone or in combination. Bar graph shows number of soft agar colonies. **D** MCF7 cells treated with leptin, adiponectin (10 µg/ml), and Honokiol (HNK) (5 µM) alone or in combination as indicated were subjected to immunoblot analysis for Med1 expression. **E**, **F** MCF7 cells were treated with leptin and 4-hydroxytamoxifen alone or combination with adiponectin or HNK as indicated and subjected to (**E**) clonogenicity assay and (**F**) soft-agar colony formation assay. **G** MCF7 cells-derived tumors were developed in nude mice and treated with Leptin, HNK and Combination. At the end of 6 weeks, tumors were collected and tumor samples (*n* = 3/group) were subjected to immunoblot analysis using Med1 and pMed1 antibodies. **H** Tumor lysate (*n* = 3/group) from MCF7-derived lean mice and obese mice treated with HNK were subjected to immunoblot analysis using Med1 and pMed1 antibodies. **I** MCF7 cells-derived tumors were developed in nude mice and treated with adiponectin, leptin alone and in combination. After 6 weeks, tumors were collected and tumor lysate (*n* = 3/group) were subjected to immunoblot analysis using Med1 and pMed1 antibodies. Actin is used as loading control. **J** Immunohistochemical (IHC) analysis for Med1 in MCF7-derived tumors treated with HNK, adiponectin (AdipoQ) as indicated. Scale bar: 50 µm. **K** Kaplan–Meier plots of breast cancer recurrences in relation to high miR-205 + low PPARBP. **L** Kaplan–Meier plots of breast cancer recurrences in relation to high miR-205 + low PPARBP + low Leptin. **M** Schematic diagram showing the signaling pathways activated in normal leptin and hyperleptinemic state. ***p < 0.0005, **p < 0.005, *p < 0.05. Data are means ± SD from three experiments.

We further explored whether Med1 is the key node by which leptin interferes with tamoxifen-mediated growth inhibition of breast cancer cells. Towards this, stable pools of MCF7^pLKO.1^ (vector control) and MCF7^shMed1^ (Med1 silenced) cells were selected in puromycin. MCF7^shMed1^ cells showed efficient silencing of Med1 in comparison with MCF7^pLKO.1^ cells (Fig. 6A). Leptin exposure induced whereas tamoxifen treatment decreased the clonogenic potential and anchorage-independent growth of MCF7 cells. Interestingly, leptin exposure alleviated tamoxifen mediated growth inhibition in MCF7^pLKO.1^ cells but no effect of leptin on tamoxifen efficacy was observed in MCF7^shMed1^ cells (Fig. 6B, C). These results indicated that Med1 is integral for leptin-mediated abrogation of tamoxifen effectiveness. Previous studies from our lab have shown that biological effect of hyperleptinemic obese state on breast cancer can be inhibited with adipocytokine adiponectin^31^ and bioactive compound honokiol^32,33^, agents known for their anti-breast cancer potential^34,35^. We questioned whether adiponectin and/or honokiol can inhibit Med1 in leptin-exposed breast cancer cells. Both honokiol and adiponectin reduced Med1 expression in leptin-exposed cells compared to leptin-alone group (Fig. 6D). In addition, honokiol and adiponectin abrogated the effect of leptin on tamoxifen effectiveness. As expected, breast cancer cells exposed with leptin showed increased clonogenicity and soft-agar colonies even in the presence of tamoxifen treatment. Interestingly, adiponectin and honokiol treatment alleviated the interfering effect of leptin, resulting in effective growth inhibition in the presence of tamoxifen (Fig. 6E, F). Analysis of breast cancer cells-derived tumors from mice treated with leptin, honokiol or a combination of leptin and honokiol showed higher expression of Med1 and phosphorylated Med1 in tumors from leptin-treated mice in comparison to honokiol-treated mice. Honokiol treatment reduced the expression of Med1 in tumors from leptin-treated mice (Fig. 6G). Furthermore, increased Med1 expression was noted in MCF7 cells-derived tumors formed in obese mice in comparison to lean mice. Honokiol treatment could inhibit Med1 expression in tumors formed in lean as well as obese mice. Immunohistochemical analysis of tumors corroborated these findings (Fig. 6H, J). Also, mice treated with adiponectin showed reduced levels of Med1 in MCF7 cells-derived tumors while leptin-treated group exhibited higher Med1 expression which is alleviated with simultaneous treatment with adiponectin. Immunohistochemical analysis of tumor samples from control, leptin, adiponectin or leptin + adiponectin treated mice showed similar findings (Fig. 6I, J). Together, these results show that Med1 is the key node for mediating anti-tamoxifen effects of leptin, and blocking Med1 with adiponectin or honokiol can alleviate the negative effects of leptin and reinstate the effectiveness of tamoxifen in hyperleptinemic state. Next, we interrogated the association of miR-205, Med1 (PPARBP) and leptin with overall survival of breast cancer patients. High expression of miR-205 and low expression of Med1 associated with better overall survival in breast cancer patients with estrogen receptor positive breast cancer (Fig. 6K). We also noted that breast cancer patients with estrogen receptor positive breast cancer exhibited better overall survival with higher miR-205, low Med1 and low leptin expression levels (Fig. 6L).

## Discussion

Although luminal A (Hormone receptor^+^/Her2^−^), a breast cancer subtype accounting for ~70% of all breast cancers, has the highest 5-year survival rate among all subtypes, it is disparately affected by obese state. Obese women with luminal A breast cancer exhibit 1.8-fold (95% CI 1.3–2.5) higher risk of breast cancer related mortality while no significant associations are observed with Her2-overexpressing or triple-negative tumors^36^. Inferior disease-free and overall survival rates are noted in obese (BMI, ≥30 kg/m^2^) and overweight (BMI, ≥25–29.9 kg/m^2^) women with luminal A breast cancer despite being treated with standard endocrine-based therapy^3^. While most studies support an association between obesity and breast cancer and report a 1.25- to 2.5-fold increase in breast cancer recurrence with increased BMI^37,38^, obese women with lymph node-negative, ER-positive breast cancer did not show increased recurrence risk and poor response to tamoxifen compared to their lean counterparts^39^. In this clinical trial, inclusion of women with operable tumors and negative axillary lymph nodes only might have led to these results as the study population did not represent a wider spectrum of disease. Nonetheless, the association between luminal A breast cancer and obesity from clinical studies^3,36^ support our observation that obese mice exhibit increased tumor progression which is not alleviated upon tamoxifen treatment. Obese state is a host-centric factor that can affect tumor growth and progression via adipocytokine dysregulation. We found that obese/hyperleptinemic tumor bearing mice did not respond to tamoxifen treatment and showed increased tumor progression implying that leptin renders breast tumors refractory to tamoxifen. This observation is supported by a recent study showing that lower levels of circulating leptin achieved via periodic fasting or a fasting mimicking diet potentiates tamoxifen efficacy^40^. Leptin expression correlates with poor prognosis in breast cancer patients treated with tamoxifen^41^. In addition, leptin signaling induces the expression of genes related to chemoresistance and its inhibition leads to chemo-sensitization^42^. Particularly significant is our finding that leptin mediates ligand-independent activation of ER, which then responds poorly to tamoxifen-directed gene repression, and exhibits reduced binding to corepressors proteins, NCoR, SMRT, and Mi2, interfering with tamoxifen-directed ER-corepressors-complex formation. Mechanistic evaluations identify the involvement of mediator protein Med1 whose inhibition leads to blocking leptin’s inhibitory effects on tamoxifen efficacy underscoring its biological importance.

It is known that phosphomodifications of ER can modulate ligand-binding, transactivation-function and response to estrogen-stimulation as well as endocrine therapy^22^. Phosphorylation of ER ensues conformation changes that pose steric hindrance to antiestrogen binding and associated cofactor recruitment^22,43^. We observed that leptin increases ER phosphorylation at serine residues 118 and 167 in breast cancer cells while no phosphorylation was observed at Ser-102/104/106, Ser-282, Ser-305, and Tyrosine-537 (null data not shown). S118 and S167 phosphorylation events increase the affinity of ER to estrogen and coactivator binding while reducing its affinity for tamoxifen^43^. Indeed, leptin exposure reduced the recruitment of corepressors to ER even in the presence of tamoxifen. Breast cancer patients harboring ER-S118 show poor response to tamoxifen and worse prognosis. On the other hand, ER-S118 may not influence breast cancer progression in patients not treated with endocrine therapy^44^. Clinical significance of ER-S167 is unclear as it is associated with poor prognosis for primary tumors but correlates with longer survival in metastatic disease^45^. It is noteworthy that ER phosphorylation at S118 and S167 can be directed by MAPK, EGFR, and Akt which are integral components of canonical leptin signaling pathway. Our data shows that leptin stimulates ER phosphorylation leading to reduced binding of corepressors complexes and abrogation of tamoxifen-mediated gene repression.

Med1 (also known as DRIP205, PBP, and TRAP220) is one of the anchor subunits of Mediator complex, an evolutionary conserved multi-subunit complex consisting of ~25 subunits which is required for the recruitment of the RNA polymerase II-complex to facilitate gene transcription. Med1 is essential for maintaining estrogen-ER function in normal physiological pubertal mammary gland development and luminal progenitor/stem cell differentiation in vivo as well as regulating the expression of ER-responsive genes in breast cancer^24,46^. Interestingly, Med1 is overexpressed in ~40–50% of breast cancers^28^ and associates with poor clinical response in breast cancer patients treated with endocrine therapy. These clinical observations are supported by preclinical studies showing that Med1 plays a significant role in response and resistance to endocrine therapy^47^. Our study implicates Med1 as an important coactivator mediating the biological effects of obesity, as 48 out of 74 genes coining the obesity-associated gene signature are putative targets for Med1 recruitment. Indeed, we observed elevated Med1 expression in leptin-treated breast cancer cells and breast tumors developed in obese mice. We found that leptin upregulates Med1 expression via inhibiting miR-205, whose overexpression associates with poor prognosis in ER-positive breast cancer patients. Phosphorylation of Med1 has been shown to enhance its coactivation function by promoting its interaction with mediator complex and nuclear hormone receptors^29,47^. Our results show that leptin increases Med1 phosphorylation via activation of Her2 and EGFR kinases, whose inhibition not only blocks nuclear accumulation of Med1 but also its interaction with ER upon leptin stimulation. Since our results demonstrate that Med1 activation is key to mediating leptin’s inhibitory effects on tamoxifen efficacy, implication of Her2 and EGFR in leptin-mediated Med1 activation opens up an opportunity to utilize Her2 and EGFR inhibitors to abrogate leptin.

Our results advance the understanding of the molecular mechanisms underlying obese hyperleptinemic state and associated poor response to tamoxifen. In this study, we present miR205 as a regulator of Med1, as well as decipher a ‘phospho-switch’ for Med1 which is regulated by ErB kinases Her2-EGFR, in leptin stimulated breast cancer. Although discovery of leptin-miR205-Med1 and leptin-Her2-EGFR-Med1 axes presents various approaches to inhibit Med1 in obese hyperleptinemic state using small molecule inhibitors, we examined whether bioactive/adipocytokine based modes can be beneficial. Indeed, breast tumors developed in obese hyperleptinemic mice treated with Honokiol, a bioactive compound isolated from Magnolia plants, exhibit reduced Med1 expression. Previous studies from our lab have shown that oral administration of honokiol inhibits breast cancer^33^. Adiponectin is a guardian angel adipocytokine with anti-breast cancer potential, whose expression is reduced in obese state^31,35,48^. Adiponectin administration in breast tumor-bearing mice exposed to leptin results in reduced Med1 expression in breast tumors indicating that strategies to elevate adiponectin level in obese state can be beneficial. Various strategies including direct upregulation of adiponectin function using ADIPOR agonists or indirect approaches targeting adiponectin-signaling molecules AMPK and PPARG/PPARγ (peroxisome proliferator activated receptor gamma) with AMPK activators and PAPAR agonists including ciglitazone, rosiglitazone, pioglitazone and troglitazone^48,49^ can be potentially utilized to inhibit Leptin-Med1 axis. Direct targeting of coactivator protein is challenging but a recent study showed successful targeting of Med1 with biosafe pRNA–HER2apt–siMED1 nanoparticle^50^. Similar strategies could be developed to target Med1 in hyperleptinemic state.

Hyperleptinemic obese state results in a ligand-independent activation of ER which recruits Med1 coactivator to EREs even in the presence of tamoxifen resulting in an elevated expression of ER-responsive genes. Leptin, not only supports an elevated expression of Med1 via inhibiting miR-205, but also induces ErbB kinase-mediated phosphorylation of Med1 which potentiates its nuclear localization and transactivation function (Fig. 6M). Our studies implicate leptin-miR205-Med1 and leptin-Her2-EGFR-Med1 axes by which obese/hyperleptinemic state may contribute to poor response to tamoxifen.

## Methods

### Cell culture and reagents

Human breast cancer cell lines MCF7, BT474 and T47D, purchased from American Type Culture Collection (ATCC), were thawed from early passage liquid nitrogen vapor stocks as required and maintained at 37 °C in 5% CO_2_ and 95% humidity. TamR (MCF7-OHT cells) cells were previously developed by our group^47^. TamR (MCF-OHT) cells show tamoxifen resistance and exhibit no change in ER expression from parent cells^47^. Cell lines are routinely authenticated with STR profiling. Cells were grown in dextran charcoal stripped media and phenol red free media prior to stimulation with leptin. Antibodies for PCNA (13–3900; 1:1000 dilution) was procured from Thermo Fisher Scientific (Waltham, MA). Antibodies for phosphorylated ER-ser 118 (2511; 1:500 dilution), ER-ser 167 (64508; 1:500 dilution), ERK (9102; 1:1000 dilution), phosphorylated-ERK (9101; 1:1000 dilution), EGFR (2232; 1:1000 dilution), phosphorylated EGFR (3777; 1:1000 dilution), Her2 (2248; 1:1000 dilution), phosphorylated Her2 (2247; 1:1000 dilution), c Myc (5605; 1:1000 dilution), cyclin D1 (2978; 1:1000 dilution), TFF1 (15571; 1:1000 dilution) were obtained from Cell Signaling Technology (Danvers, MA). Med1 antibody (SC-5334; 1:500 dilution), Cathepsin D (SC-6486; 1:500) and anti-goat secondary antibody (SC-2922; 1:5000) were purchased from Santa Cruz Biotechnology, Inc. (Dallas, Texas). Phosphorylated Med1 antibody (ab64965; 1:1000) was procured from Abcam (Cambridge, UK). Leptin, Adiponectin, 4-Hydroxy-Tamoxifen, estradiol and antibodies targeting β-actin (A54441; 1:5000), anti-mouse secondary (NA931V; 1:5000 dilution), and anti-rabbit secondary (NA934V; 1:5000 dilution) were purchased from Sigma-Aldrich (St. Louis, MO). Inhibitors of epidermal growth factor receptor (AG1478) and ERBB2 (AG825) were purchased from Calbiochem/Millipore Sigma (Burlington, MA). Mimic and inhibitor for miR-205 were purchased from ThermoFisher Scientific (Waltham, MA). Honokiol (HNK) is a natural product extracted from the seed cone of *Magnolia grandiflora* via HPLC with 98% purity^51^.

### Obese mice model and breast tumorigenesis assay

All animal studies were approved by the Johns Hopkins ACUC. NOD/SCID mice (female, 6–8 weeks old) were acquired from SKCCC animal facility and maintained in-house. Mice were fed a 60% kcal fat diet (D12492i, Research Diets Inc., New Brunswick, NJ, USA) to induce diet-induced obesity (DIO) or a 10% low-fat control diet (12450Bi, Research Diets Inc., New Brunswick, NJ, USA) for ~12 weeks ad libitum before the experiment and the high-fat-diet (HFD) and control normal diet (ND) groups were maintained on respective diets throughout the experiment. Estrogen pellets (60-day slow-release pellet containing 0.72 mg of 17β-estradiol; Innovative Research of America) were subcutaneously implanted 3 days prior to tumor cell inoculation for sustained estrogen supplementation. Exponentially growing MCF7 (5 × 10^6^ cells in 100 μl matrigel), were implanted in the 4^th^ mammary fat pad on either side. Vehicle and tamoxifen treatment (500 μg of tamoxifen citrate in peanut oil by subcutaneous injection daily Monday to Friday, for 6 weeks) were given to respective groups. *For leptin-treated group*, mice were treated with recombinant leptin (i.p., dosage of 5 mg/kg), 5 days a week for the duration of the experiment. Mice were regularly monitored for tumor incidence and tumor growth was measured twice a week using calipers for 6 weeks. Tumors were excised and processed for further analysis. *For immunohistochemistry*, tumors were fixed in 10% formalin, paraffin embedded and sectioned. Tissue sections were deparaffinized and rehydrated followed by antigen retrieval in citrate buffer. IHC for specific proteins was carried out using specific antibodies and Vectastain ABC detection system (Vector Laboratories, Burlingame, CA, USA) according to Vector lab guidelines. Sections were counter stained with hematoxylin, dehydrated and mounted followed by image analyses using Lieca microscope at ×20 magnification. *Adiponectin and honokiol treatment:* Mice were treated with adiponectin (intratumoral injections of recombinant adenovirus [10^8^ plaque-forming units (pfu)] expressing adiponectin, thrice a week), and/or Honokiol (3 mg/mouse/day in 20% Intralipid [Baxter Healthcare, Deerfield, IL], three times per week) for the duration of the experiment.

### Clonogenicity, soft-agar colony-formation assay

For *clonogenicity* assay, cells were treated, counted and plated in 12-well plates. Colony numbers were assessed using ImageJ after fixing and staining the colonies with crystal violet (0.1% in 20% methanol) (Sigma-Aldrich, C3886). For soft-agar colony-formation assay, 1 × 103 cells were suspended in 1:1 mix of 0.6% agar and complete medium containing treatments and added to 6-well plates containing solidified 0.6% base agar followed by incubation for 3 weeks. Colonies were stained with 0.005% crystal violet and observed using inverted microscope (Eclipse Ti2, Nikon, Melville, NY). Colonies were counted in five randomly selected fields at 10x magnification.

### Whole cell lysates, nuclear-cytoplasmic fractionation and immunoblotting

*Whole* cell lysates of breast cancer cells were prepared using modified RIPA buffer^52^. Nuclear-cytoplasmic lysates were prepared using NE-PER^TM^ Nuclear and Cytoplasmic Extraction Reagents (Thermo Fisher Scientific, Waltham, MA) according to the manufacturer’s protocol. Equal amounts of whole cell lysates, nuclear extracts and cytoplasmic extracts were subjected to immunoblot analysis^34^. All blots are derived from same experiments and were processed in parallel.

### Phosphokinase array, immunofluorescence, and confocal imaging

*Phosphokinase analysis* was performed using the Proteome Profiler Human Phospho-Kinase Array Kit ARY003 (R&D Systems)^53^. Array images were analyzed using the GeneTools image analysis software (Syngene). For Immunofluorescence, breast cancer cells (5 × 103) were seeded on chambered slides, allowed to grow for 24 h and treated as indicated and subjected to immunofluorescence analysis^34^. Immunofluorescently stained cells were imaged using a Zeiss LSM510 Meta (Zeiss, Dublin, California, USA) laser scanning confocal system configured to a Zeiss Axioplan 2 upright microscope (Zeiss, Dublin, CA, USA).

### RNA isolation, RT-PCR, transfection and chromatin immunoprecipitation assay

For *RT-PCR*, cells were lysed in Trizol, RNA was isolated by chloroform-isopropanol method and cDNA was synthesized using iScript cDNA synthesis kit followed by reverse transcription-polymerase chain reaction (RT-PCR)^34^. For *miR assays*, cells were transfected with miR-205 mimic or miR-205 inhibitor or control-miR (Applied Biosystems, Ambion, Austin, TX) using Fugene transfection reagent (Promega Corporation, Madison, WI). For qRT-PCR detection of miR-205, miRNA-specific RT-primers, TaqMan miRNA Assay (Applied Biosystems, Ambion, Austin, TX) and Platinum Taq Polymerase Reagents (Invitrogen, Grand Island, NY) were used. Data were calculated by using the standard ΔΔ*Ct* method and microRNA expression was represented as fold-difference of each treatment *vs*. vehicle-treated control. Chromatin immunoprecipitation (ChIP) analyses were conducted using our published procedure. Briefly, chromatin lysates were prepared and subjected to immunoprecipitation using specific antibodies^34^. Following primers were used. Cathepsin D-RT-PCR: Forward primer: 5ʹ-CCA GCC CCC AAT CCC AAC CCC ACC TCC AG-3ʹ, Reverse primer: 5ʹ-CAC TGA AGC TGG GAG GCA AAG GCT ACA AGC-3. c-Myc-RT-PCR: Forward primer: 5ʹ-GCC ACG TCT CCA CAC ATC AG-3ʹ, Reverse primer: 5ʹ-TCT TGG CAG CAG GAT AGT CCT T-3. EBAG9-RT-PCR: Forward primer: 5ʹ-GCT ACA CAA GAT TCT GCC T-3ʹ, Reverse primer: 5ʹ-CTT CTT CAT TAG CCG TTG TG-3ʹ. XBP1-RT-PCR: Forward primer: 5ʹ-CCT TGT AGT TGA GAA CCA GG-3ʹ, Reverse primer: 5ʹ-GGG GCT TGG TAT ATA TGT GG-3ʹ. TFF1-RT-PCR: Forward primer: 5ʹ-TTT GGA GCA GAG AGG AGG-3ʹ, Reverse primer: 5ʹ-TTG AGT AGT CAA AGT CAG AGC AG-3. β-actin-RT-PCR: Forward primer 5ʹ-ACC ATG GAT GAT GAT ATC GC-3ʹ, Reverse primer: 5ʹ-ACA TGG CTG GGG TGT TGA AG-3ʹ. Cathepsin D-ChIP: Forward primer: 5ʹ-GGT TTC TCT GGA AGC CCT GTA G-3ʹ, Reverse primer: 5ʹ-TCC TGC ACC TGC TCC TCC-3ʹ. c-Myc-ChIP: Forward primer: 5ʹ-AGG CGC GCG TAG TTA ATT CAT-3ʹ, Reverse primer: 5ʹ-CGC CCT CTG CTT TGG GA-3ʹ. EBAG9-ChIP: Forward primer: 5ʹ-ATT GTC TGC CCT TCG CCG T-3ʹ, Reverse primer: 5ʹ-TTTGGAGGCTGCTGCGTGCTTT-3ʹ.

### In silico analyses

Protein-protein interactome (PPI) was constructed using NetworkAnalyst (network-based visual analytics for gene expression profiling, meta-analysis and interpretation)^54^. Key leptin signaling genes were used as input data to construct the protein-protein interactome of MED1 and its associates using STRING Interactome with a Confidence score cutoff 900^55^. Gene Set Enrichment Analysis *(*GSEA*):* Utilizing GSEA software available from the Broad Institute^56^, GSEA was performed on the data set of GSE58059^20^ and GSE26459^21^ for oncogenic pathways. Gene expression analysis of published data sets*:* microarray data analyzing gene expression of MCF7 cells treated with Leptin was searched for in NCBI-GEO database. Raw data for data set GSE58059 was retrieved by downloading the Series Matrix File. Sample information and Sequencing/Array platform information was downloaded and data was arranged as a gene expression matrix in MS Excel. Pre-processed data was then run through web based interface, integrated Differential Expression and Pathway analysis (iDEP.91). Enriched biological processes and molecular pathways were searched for using the “Pathway” application of iDEP and heat maps were constructed. For survival analyses, we performed survival analysis using the METABRIC dataset available in EGA under the study ID EGAS00000000122. The advantage of METABRIC is the simultaneous availability of mRNA and miRNA gene expression data for the same set of patients. The total number of patients with mRNA expression was 1302 and with miRNA expression 1480; all data was available for 1262 patients. Of these, 996 were ER positive when assessed by immunohistochemistry and these patients were used in the analyses. The technology used for determination of mRNA expression was Illumina HT 12 and for miRNA expression Agilent ncRNA 60k arrays. In the mRNA dataset, the probes ILLMN_1721729, ILLMN_2207505, and ILLMN_2234956 were used to determine PPARBP, LEP, and LEPR expression, respectively. Survival analysis was performed for overall survival data using Cox proportional hazards regression and by plotting Kaplan–Meier survival plots. Correlation between mRNA expression and miRNA expression was determined using Spearman rank correlation. When combining multiple genes, their mean expression was used as a signature—in this, the expression of PPARBP and LEP were inverted, because these displayed the opposite correlation to survival in the single-gene univariate analysis. The median was used as a cutoff to determine high- and low-expression cohorts. Statistical significance was set at *p* < 0.05.

### Statistical analyses

All experiments were performed thrice in triplicates. Statistical analysis was performed using Microsoft Excel software. Significant differences were analyzed using the Student *t* test and two-tailed distribution. Results were considered to be statistically significant if *p* < 0.05. Results were expressed as mean ± SE between triplicate experiments performed thrice. For animal studies, analysis of variance with repeated measurements was carried out to compare the mean tumor volume between different groups. The overall *p* value for testing for differences between at least two groups is *p* < 0.0001.

### Reporting summary

Further information on research design is available in the Nature Research Reporting Summary linked to this article.

## Supplementary information

Supplementary Material

Reporting Summary

## Data Availability

Analyzed data set GSE58059 (GEO accession number) can be accessed on NCBI, Gene Expression Omnibus https://www.ncbi.nlm.nih.gov/geo/query/acc.cgi?acc=GSE58.
