# Supplementary material for: Hyperleptinemia in obese state renders luminal breast cancers refractory to tamoxifen by coordinating a crosstalk between Med1, miR205 and ErbB
Source: NPJ Breast Cancer. 2021 Aug 13;7:105. doi: 10.1038/s41523-021-00314-9 (PMC8363746; doi:10.1038/s41523-021-00314-9)
Supplement: Supplementary file 1 — Supplementary Material [file 41523_2021_314_MOESM1_ESM.pdf]

Supplementary information

Supplementary Figure 1

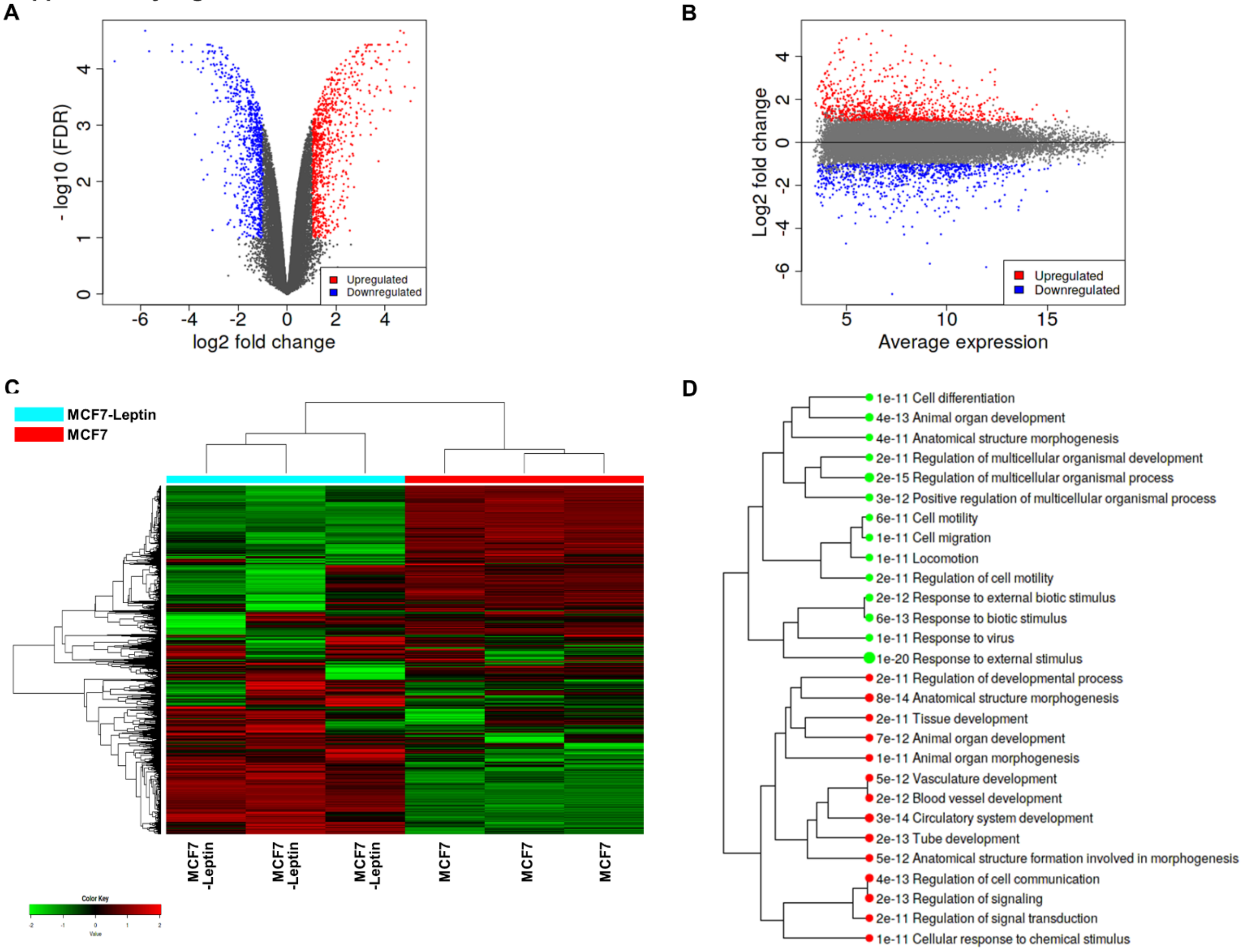

Supplementary Figure 1 *In silico* analyses of differentially expressed genes (DEGs) in MCF7 cells treated with vehicle vs. leptin. Data set GSE58059 was analyzed using iDep.91. **(A)** Volcano plot shows differential expression of genes against false discovery rate between MCF7 and MCF7-Leptin. **(B)** MA plot (M: log ratio, A: Mean average) showing average expression of genes against fold change between MCF7 and MCF7-Leptin. **(C)** Unsupervised heatmap showing top 12000 gene expression between MCF7 and MCF7-Leptin. **(D)** Tree showing upregulated and down-regulated biological processes in MCF7-Leptin compared to MCF7.

Supplementary Figure 2

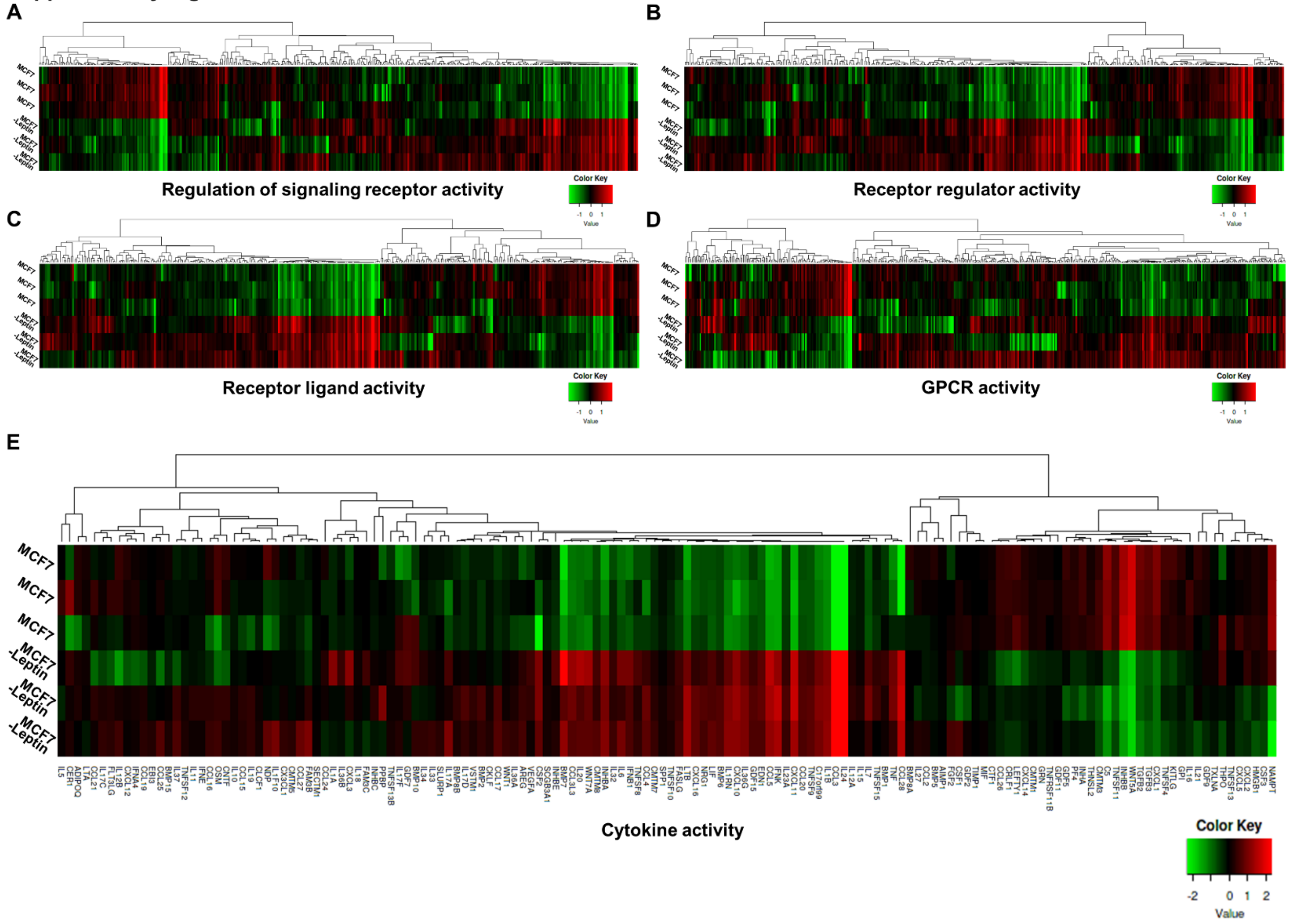

Supplementary Figure 2 *In silico* analyses of differentially expressed genes (DEGs) in MCF7 cells treated with vehicle vs. leptin. Data set GSE58059 was analyzed using iDep.91. **(A-E)** Gene ontology using pathway analysis function of iDep.91 shows significant upregulation of **(A)** Signaling receptor activity(p=2.0e-03), **(B)** Receptor regulator activity(3.1e-03), **(C)** Receptor ligand activity(2.0e-03), **(D)** G protein coupled receptor activity(7.5e-03), and **(E)** cytokine activity(1.8e-03) MCF7-Leptin compared to MCF7.

Supplementary Figure 3

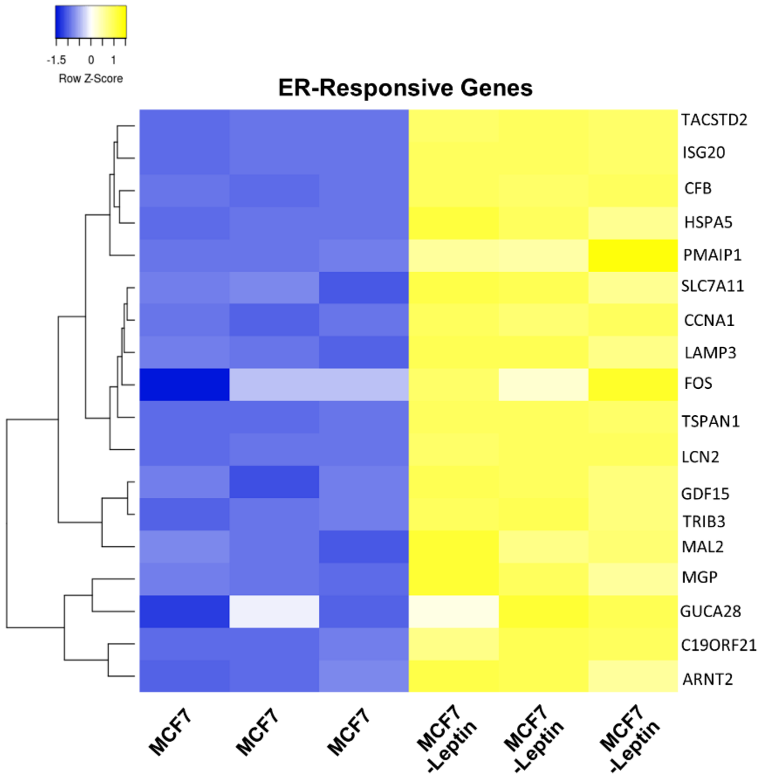

Supplementary Figure 3 *In silico* analyses of differentially expressed genes (DEGs) in MCF7 cells treated with vehicle vs. leptin. Differential gene expression analysis of data set GSE58059 using iDep.91. Supervised heatmap shows expression profile of ER-responsive target genes in MCF7 vs MCF7-Leptin cells.

Supplementary Figure 4

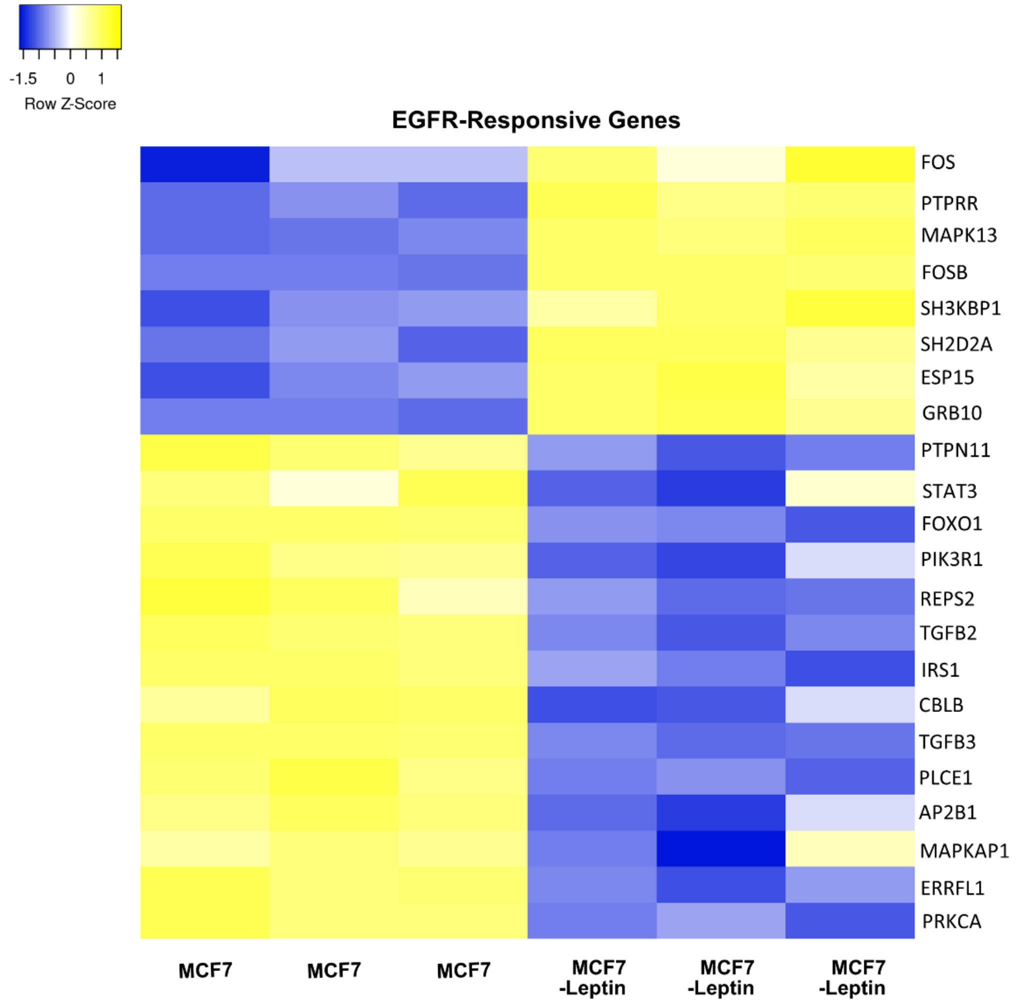

Supplementary Figure 4 *In silico* analyses of differentially expressed genes (DEGs) in MCF7 cells treated with vehicle vs. leptin. Differential gene expression analysis of data set GSE58059 using iDep.91. Supervised heatmap shows expression profile of EGFR-responsive target genes in MCF7 vs MCF7-Leptin cells.

Supplementary Table 1

Obesity Related Genes occupied by MED1									
PSPH	MRPL44	NTRK3	CDC42EP2	TBCA	CYP3A5	KCNJ13	RBBP9	MPL	DRD4
CCL18	RAD54B	VNN1	SOS1	ANTXR1	SFTPC	SRD5A2	TRPV1	CG018	MMP20
CG018	EPB41L4A	CRYBB2	APOC3	RIBC2	DLX6	SNTB2	ZNF330	BATF	C16orf34
FXYD2	WDR1	ACOX1	F2	PTTG3	ADAMTS2	CRYGC	SLC7A4	SPN	TEX13A
HRH3	RNF126	TM4SF4	SFXN1	PLD1	ZBTB7C	SPN	KIAA0652	CDH18	IGFALS
SPANXA1	LOC653580	SIT1	U2AF2	GRM8	HRBL	PGAM2	HRH3	POU3F3	IFT122
IL24	HIST1H3F	FBLN1	GTSE1	ZNF335	HSP90B1	TSP50	SPANXB1	SPANXA2	SPANXC
SPANXB2	CARKL	MB	LOC652683						

Figure 2A

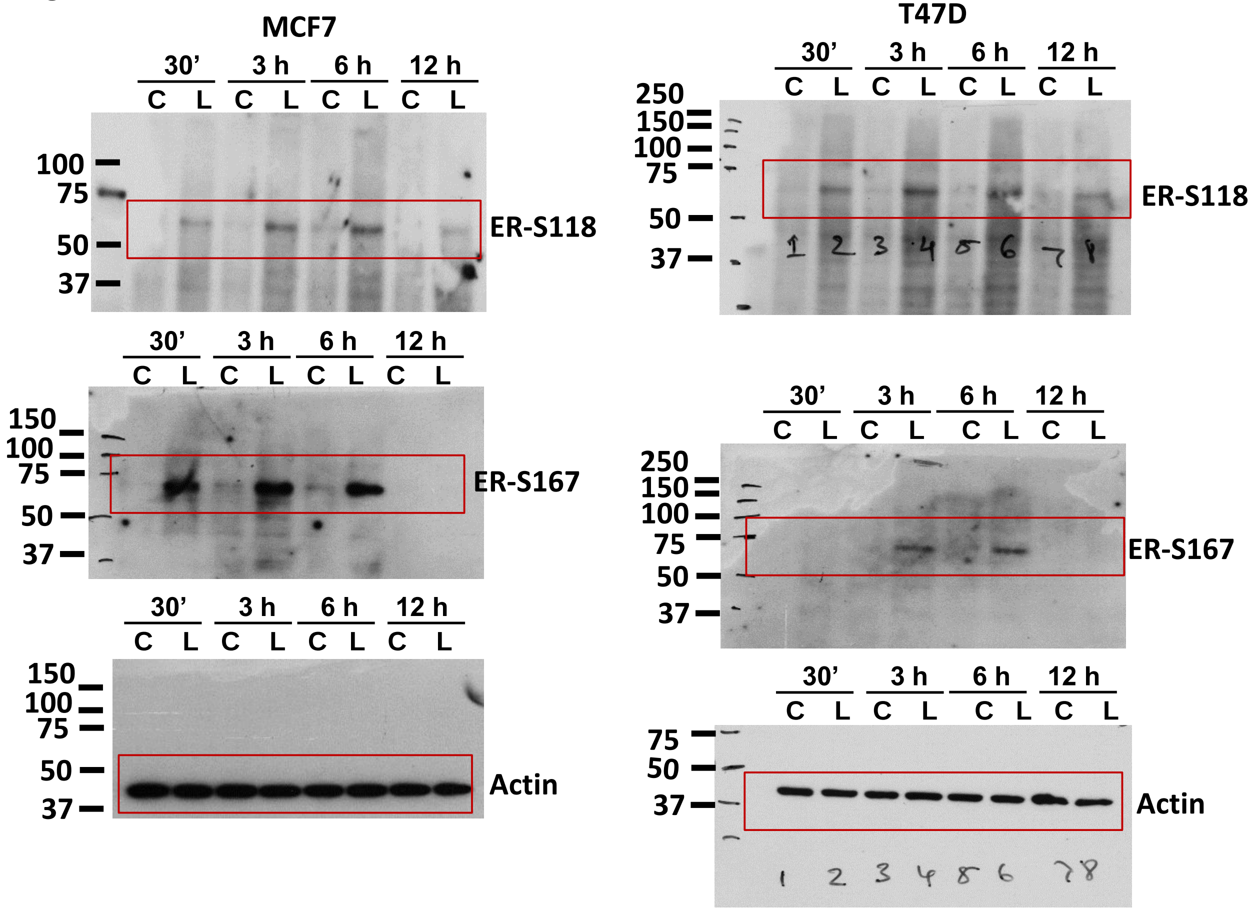
MCF7
T47D
30’
3 h
6 h
12 h
C
L
ER-S118
ER-S167
Actin
250
150
100
75
50
37
1 2 3 4 5 6 7 8

Figure 3E

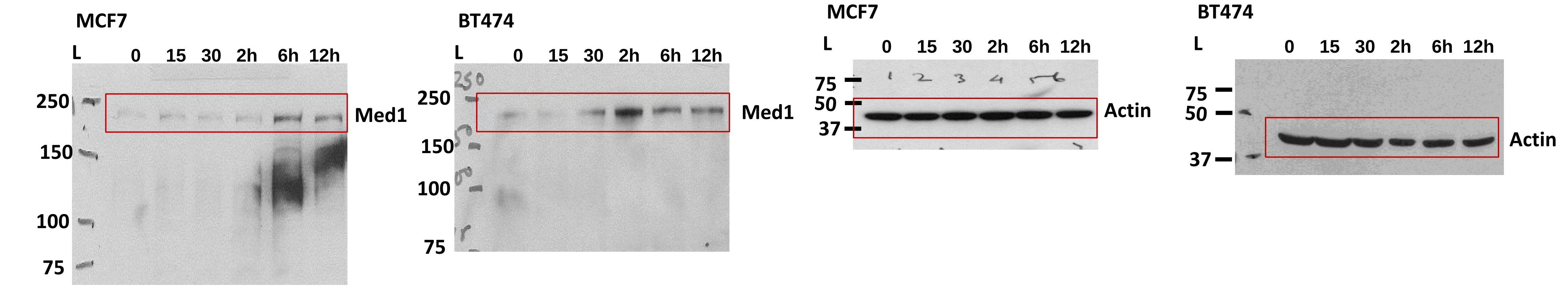

Figure 3G

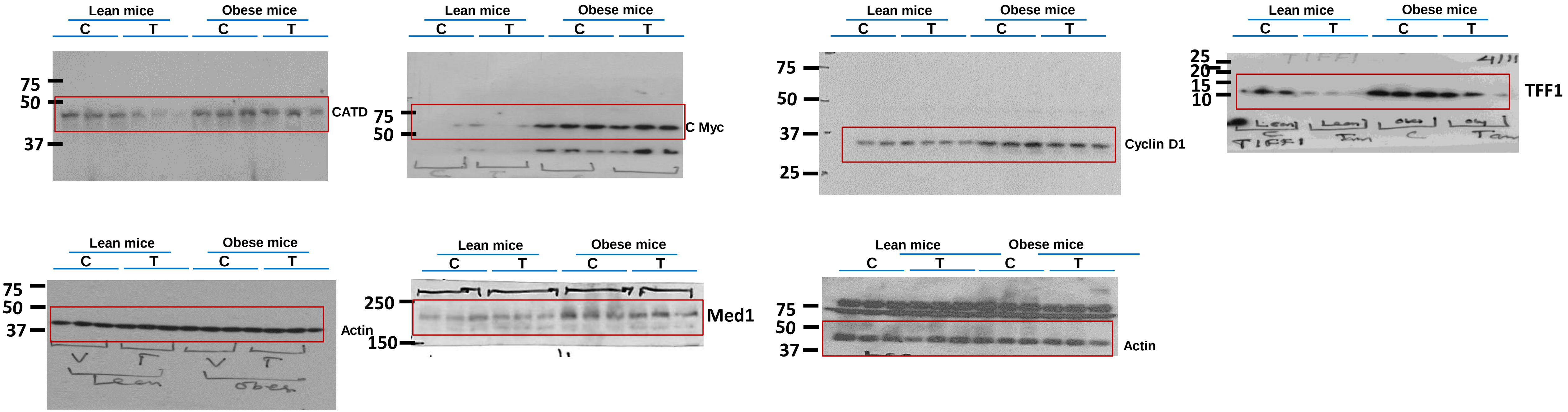

Figure 4A

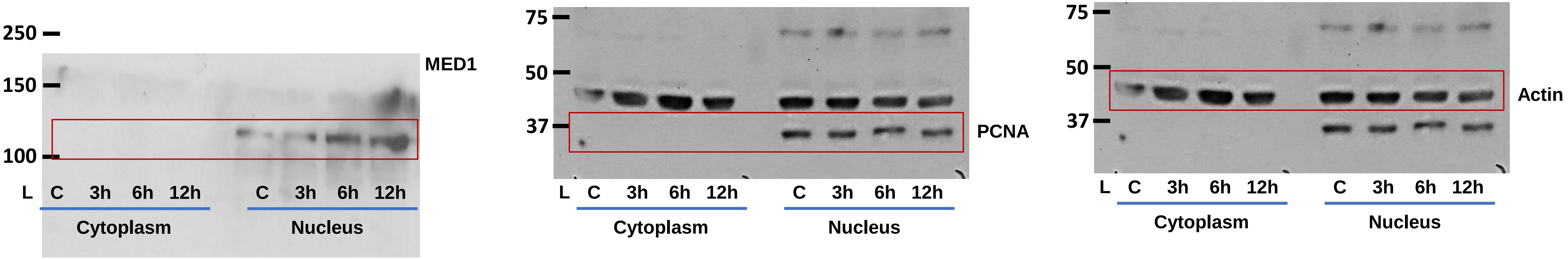

Figure 4D

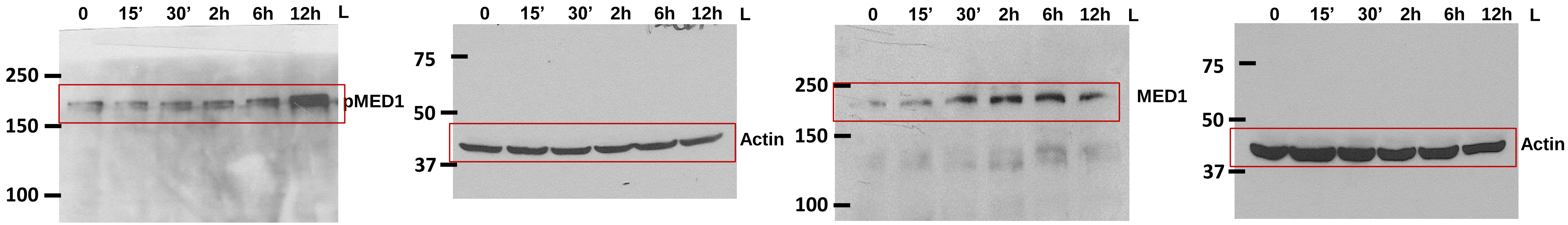

Figure 4E

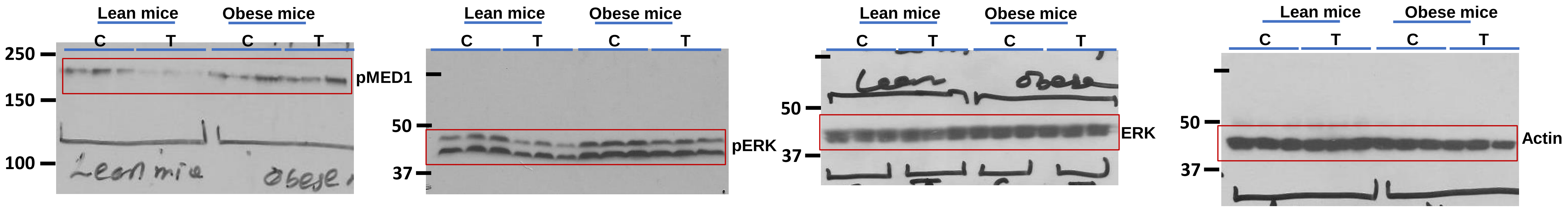

Figure 4F

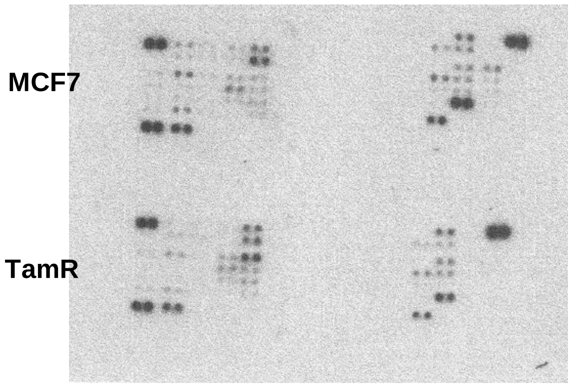

Figure 4G

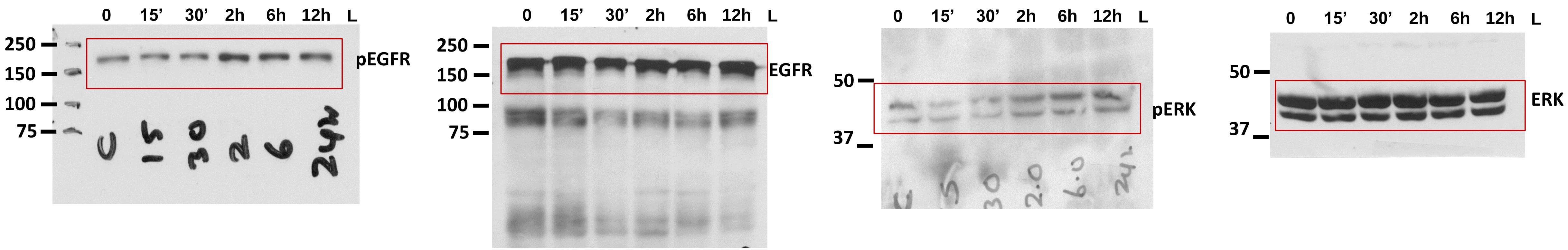

Figure 5A

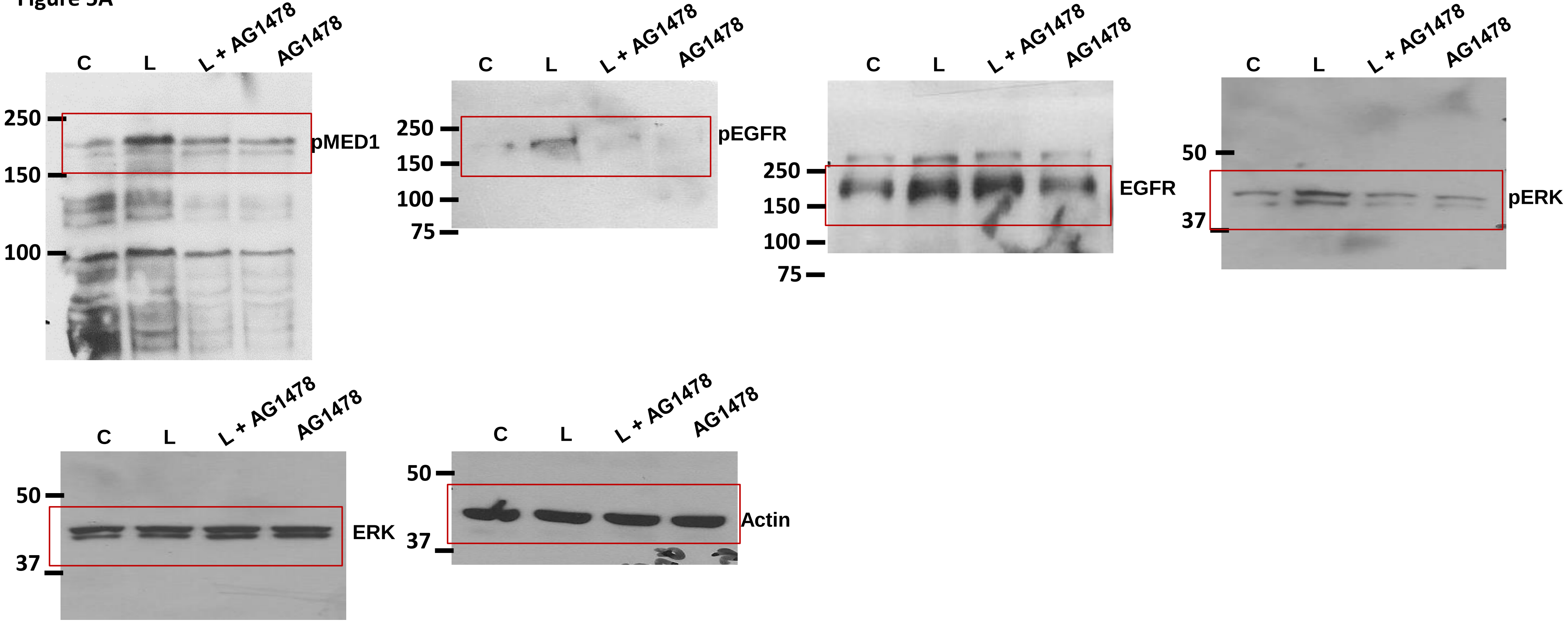

Figure 5B

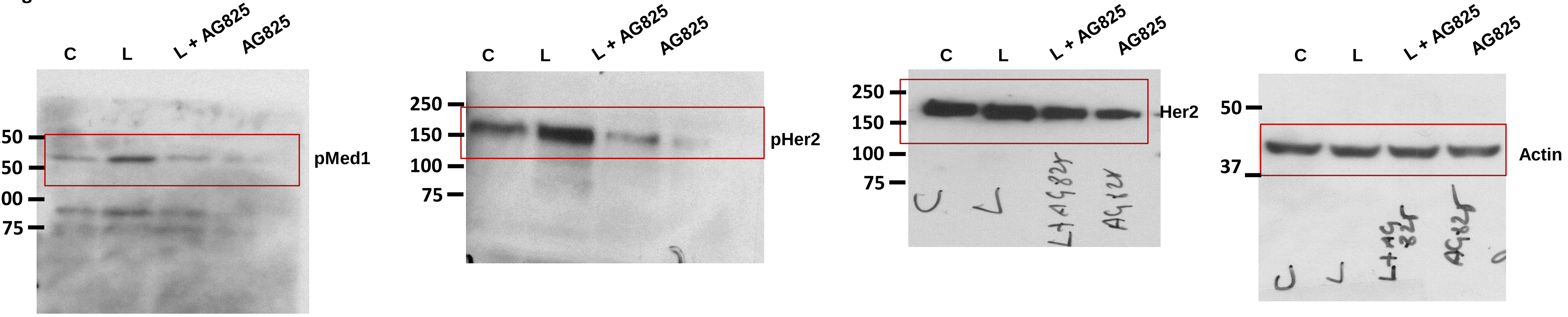

Figure 5D

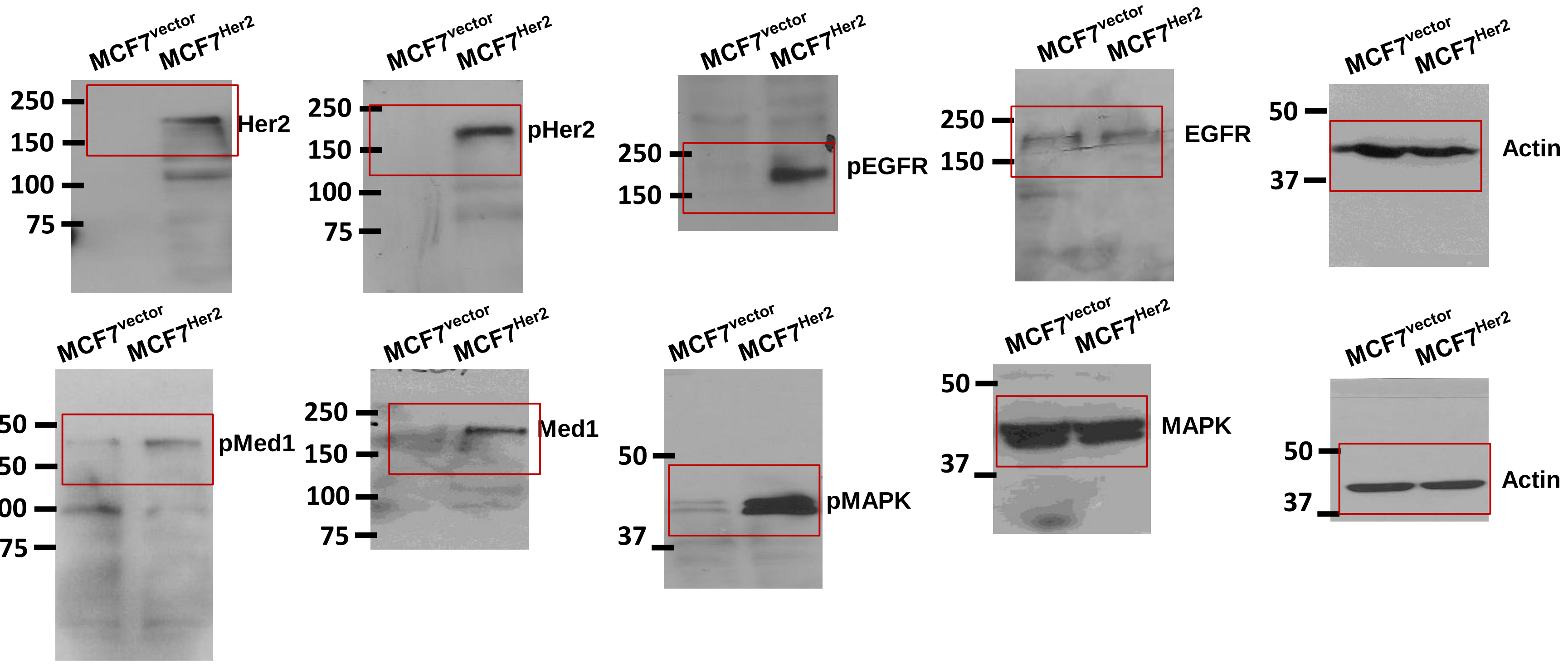

Figure 5E

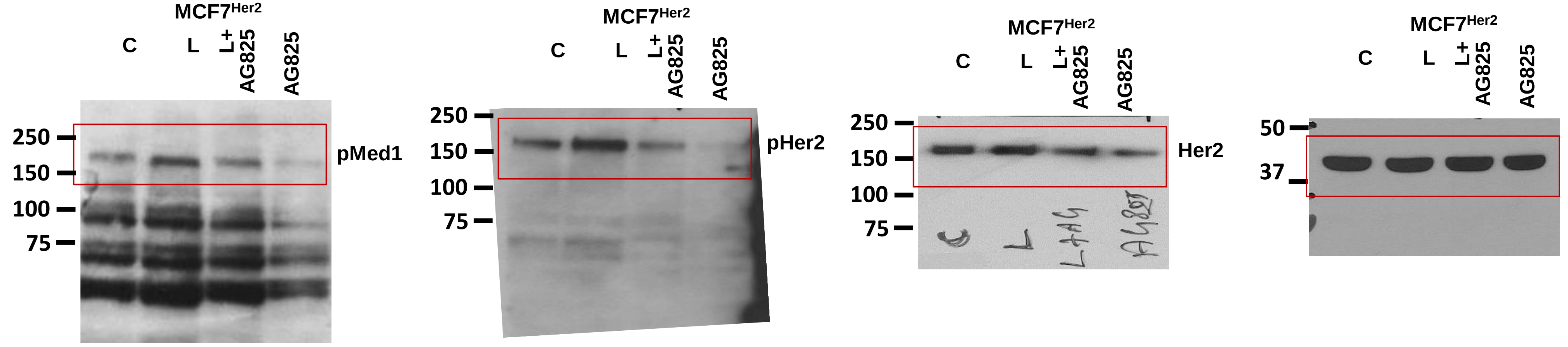

Figure 6A

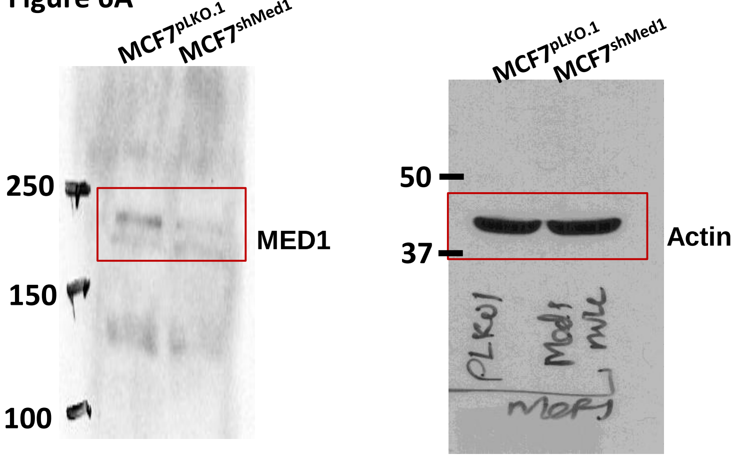

Figure 6D

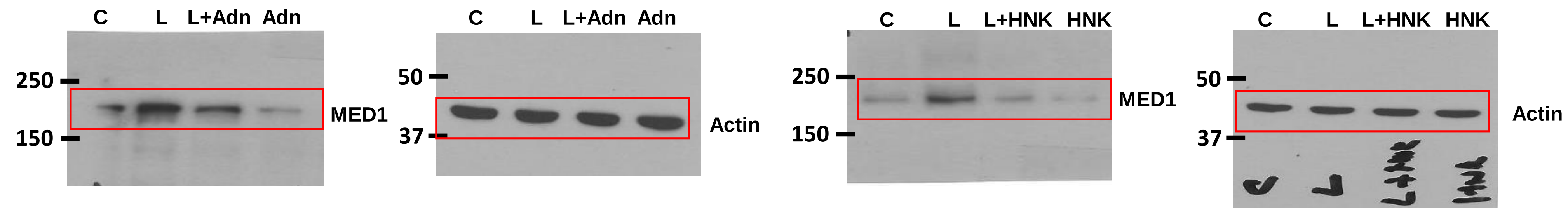

Figure 6G

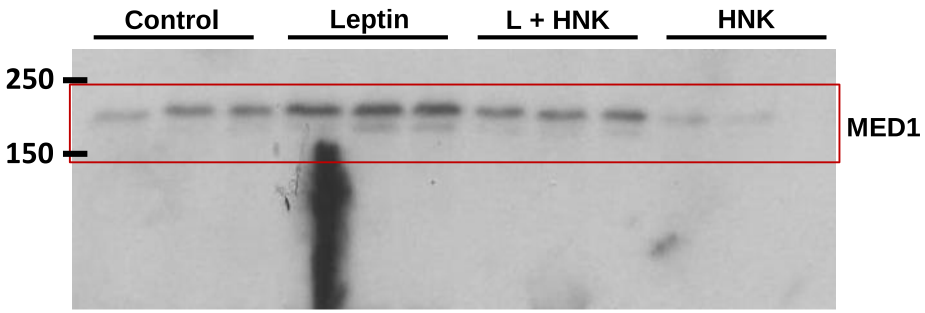

Xenograft Tumors

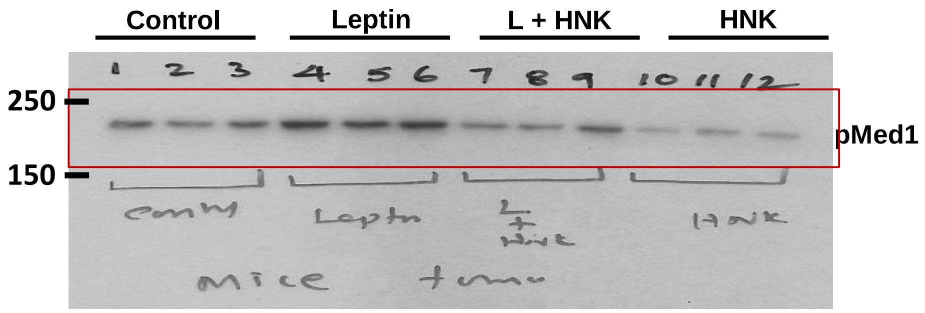

Xenograft Tumors

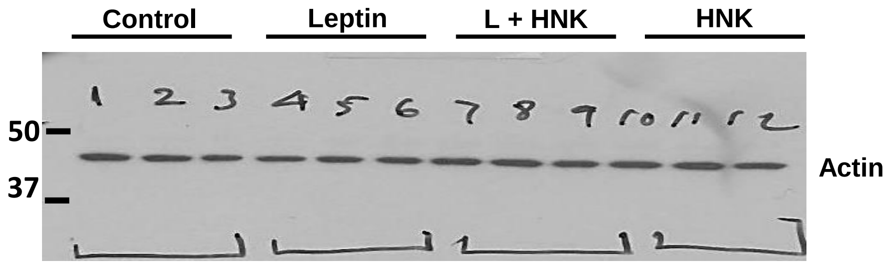

Xenograft Tumors

Figure 6H

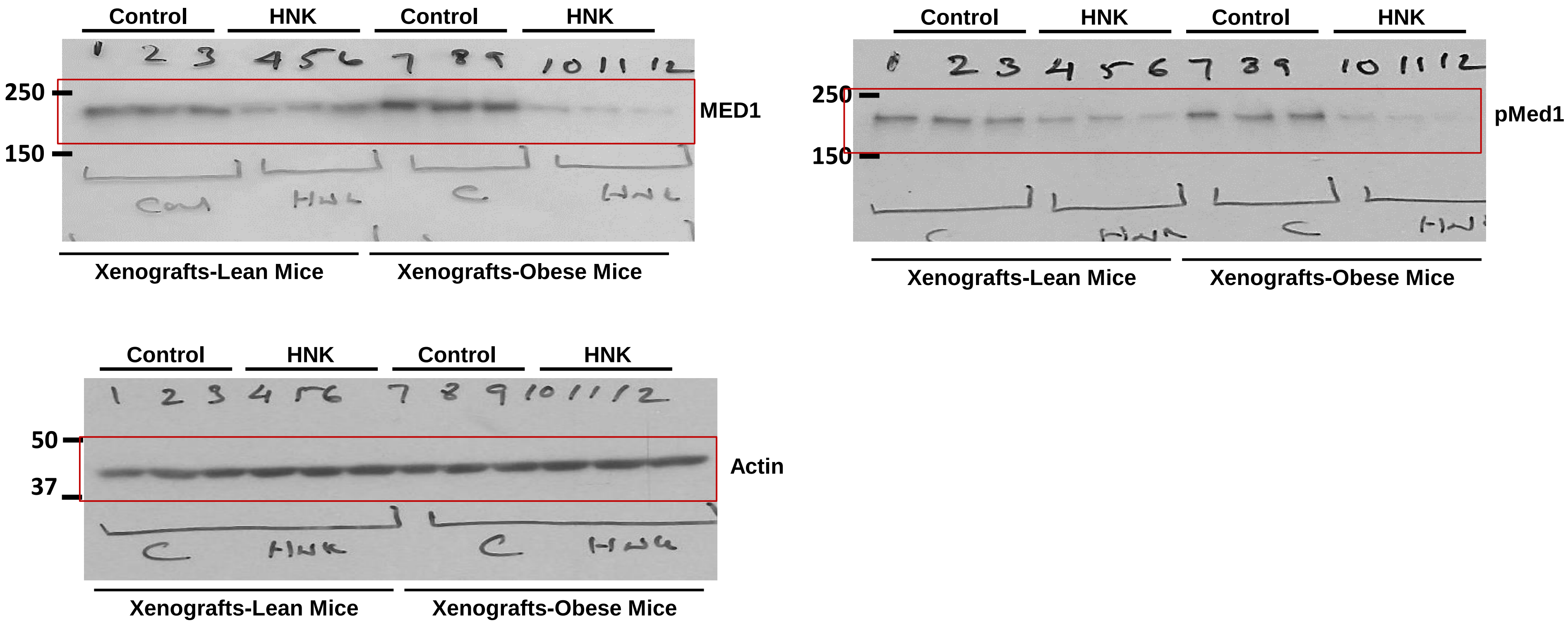
Control
HNK
Control
HNK
250
150
MED1
Xenografts-Lean Mice
Xenografts-Obese Mice
Control
HNK
Control
HNK
250
150
pMed1
Xenografts-Lean Mice
Xenografts-Obese Mice
Control
HNK
Control
HNK
50
37
Actin
Xenografts-Lean Mice
Xenografts-Obese Mice

Figure 6I

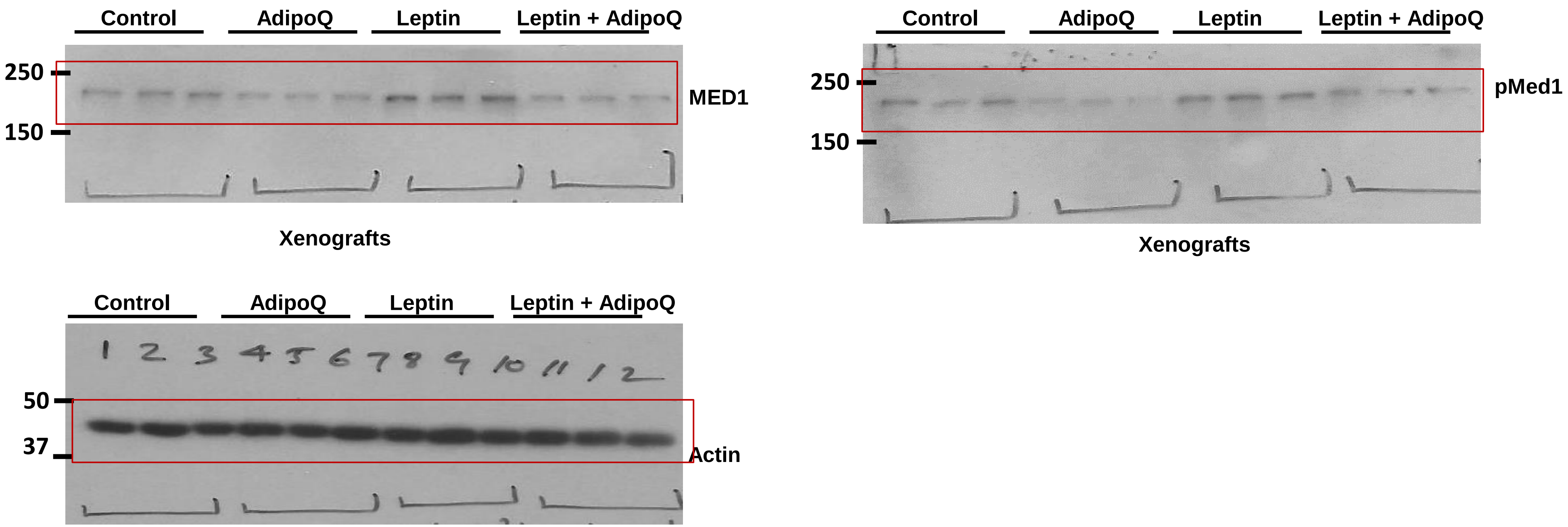
Control
AdipoQ
Leptin
Leptin + AdipoQ
250
150
MED1
Xenografts
Control
AdipoQ
Leptin
Leptin + AdipoQ
250
150
pMed1
Xenografts
Control
AdipoQ
Leptin
Leptin + AdipoQ
1 2 3 4 5 6 7 8 9 10 11 12
50
37
Actin
